# Elemental Geochemical Characteristics of Shales from Wufeng–Longmaxi Formations in the Southern Margin of the Qinling Orogenic Belt, China: Implications for Depositional Controls on Organic Matter

**DOI:** 10.1021/acsomega.4c01129

**Published:** 2024-07-15

**Authors:** Bin Xiao, Dongxu Guo, Zhongying Zhao, Shuzhen Xiong, Mingfei Feng, Zhonghai Zhao, Sheng Li

**Affiliations:** † College of Mining, Liaoning Technical University, Fuxin 123000, China; ‡ PetroChina Research Institute of Petroleum Exploration & Development, Beijing 100083, China; § College of Environmental Science and Engineering, Liaoning Technical University, Fuxin 123000, China; ∥ Liaoning Geology Engineering Vocational College, Dandong 118000, China

## Abstract

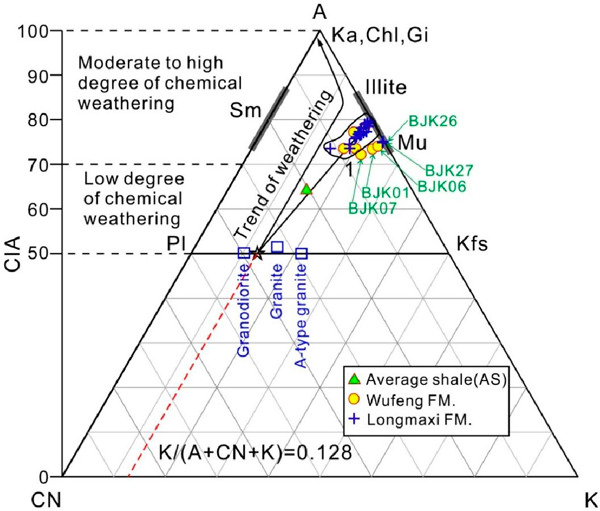

The tectonic background and sedimentary environment during the transition period from the Ordovician to Silurian have been widely studied by many scholars. This study focuses on the Upper Ordovician Wufeng Formation and Lower Silurian Longmaxi Formation in the Bajiaokou profile at the southern margin of the Qinling Orogenic Belt in southern China. In order to study the aggregation mechanism of organic matter, geochemical proxies were proposed, including redox proxies (V, V/Al, U, U/Al, Mo, and Mo/Al), paleoproductivity proxies (P, P/Ti, Ba, Ba/Al, and Si_XS_), paleoclimate proxies (CIA), and terrigenous flux proxies (Al, Zr, and Zr/Al). In addition, Al–Co[EF] × Mn[EF] is used to provide information on paleoenvironmental parameters such as watermass restriction conditions. The redox proxies show that the Wufeng–Longmaxi shale is mainly accumulated under oxic–dysoxic conditions. During the shale deposition period of Wufeng–Longmaxi formations, the marine surface primary productivity in the southern Qinling area is generally low to moderate. The paleoclimate proxies show that from the Late Ordovician to the Early Silurian the southern Qinling area generally had a warm and humid climate. The upwelling current is widely developed in the northern margin of the Sichuan Basin and the southern margin of the Qinling area. Although the upwelling current was highly developed during the deposition of the Wufeng Formation in the Bajiaokou profile, the concentrated accumulation of a large amount of volcanic ash resulted in the low primary productivity of the ocean. During the sedimentary period of the Longmaxi Formation in the Bajiaokou profile, the development of seasonal upwelling currents and a small amount of volcanic ash supply increased the primary productivity to moderate, which provided a good material basis for the enrichment of organic matter, but the high detritus flux and the water body condition of oxic–dysoxic resulted in the slight enrichment of organic matter.

## 1 Introduction

The end of the Ordovician was an important period in Earth’s history,^1−7^ which is marked by marine mass extinction, sea-level change, and extensive deposition of marine black shale.^8−14^ In recent years, with the global attention on atmospheric environmental protection, natural gas as a clean energy mineral resource has received more and more attention from many countries. As one of the large oil-bearing basins, the Sichuan Basin in south China is rich in natural gas resources and has made remarkable achievements in the exploration and development of unconventional natural gas.^15^ The Wufeng–Longmaxi formation shale is the earliest shale gas commercially developed formation in China. From 2012 to 2022, shale gas fields were discovered in the formations in south China, including Fuling in Chongqing Municipality, Weiyuan–Changning in Sichuan Province, and Zhaotong in Yunnan Province. The proven geological reserves were 10,455 × 10^8^ m^3^, and the shale gas production capacity was 200 × 10^8^ m^3^ per year.^16,17^ A large amount of geological data has been obtained from shale gas exploration and development in the eastern and southern parts of the Sichuan Basin. Chen et al.^18^ (2015) studied the development of drill core graptolite zones in shale gas demonstration areas and identified four graptolite zones from WF1 to WF4 in the Wufeng Formation and nine graptolite zones from LM1 to LM9 in the Longmaxi Formation. At the same time, six favorable shale gas blocks were proposed after studying the Wufeng–Longmaxi formations in the entire Sichuan Basin and its surrounding areas. The northern margin of the Sichuan Basin is considered to be the most stable area for the development of black shale in the Wufeng–Longmaxi formations and is one of the most promising areas for shale gas exploration and development. Nie et al.^19^ (2017) studied the outcrops and drilling conditions in the eastern and southern regions of the Sichuan Basin and found that the black shale at the bottom of the Longmaxi Formation has good shale gas enrichment conditions and has great potential for shale gas exploration and development. Nie et al.^19^ (2017) redetermined the sedimentary facies and thickness distribution at the bottom of the Longmaxi Formation in the Sichuan Basin and adjacent areas through research. The exploration of shale gas in the northern margin of the Sichuan Basin is mainly concentrated in the Chengkou–Wuxi area. The main drilling targets for the Wufeng–Longmaxi formation shale include the Yucan 1 well, Wuxi 1 well, and Wuxi 2 well. Previous studies have conducted detailed analyses on the stratigraphic development, lithological characteristics, shale pore structure characteristics, and gas bearing properties of these wells.^20−27^ However, research on the overall distribution and lithological characteristics of outcrops in the Wufeng–Longmaxi formations of the Qinling Orogenic Belt on the northern margin of the Sichuan Basin is relatively weak.

Therefore, the study on the stratigraphic development characteristics and sedimentary environment of the Upper Ordovician Wufeng Formation and the Lower Silurian Longmaxi Formation in the southern margin of the Qinling Orogenic Belt, as well as the mechanism of organic matter accumulation, can not only provide an important understanding of the evolution of the paleoclimate and paleoenvironment in this area but also provide a basis for the exploration and development of shale oil and gas resources.

Some geochemical parameters can be used to characterize the sedimentary environment of shale by analyzing the geochemical characteristics of the whole shale rock.^28−32^ In addition, Al–Co[EF] × Mn[EF] can be used to discuss information about paleoenvironmental parameters such as watermass restriction conditions.^33^ The main purpose of this study is to reconstruct the sedimentary environment of the Wufeng–Longmaxi formations on the southern margin of the Qinling Orogenic Belt and to discuss the main factors affecting the production and preservation of organic matter during the deposition of these shale sequences. This study mainly includes the following aspects: (1) the developmental characteristics and geochemical characteristics of the shale of Wufeng–Longmaxi formations in the study area; (2) source input, climate change, seawater redox conditions, marine primary productivity, and watermass restriction during shale deposition; and (3) the main controlling factors of organic matter accumulation in shale.

## 2 Geological Setting

**Figure 1:**
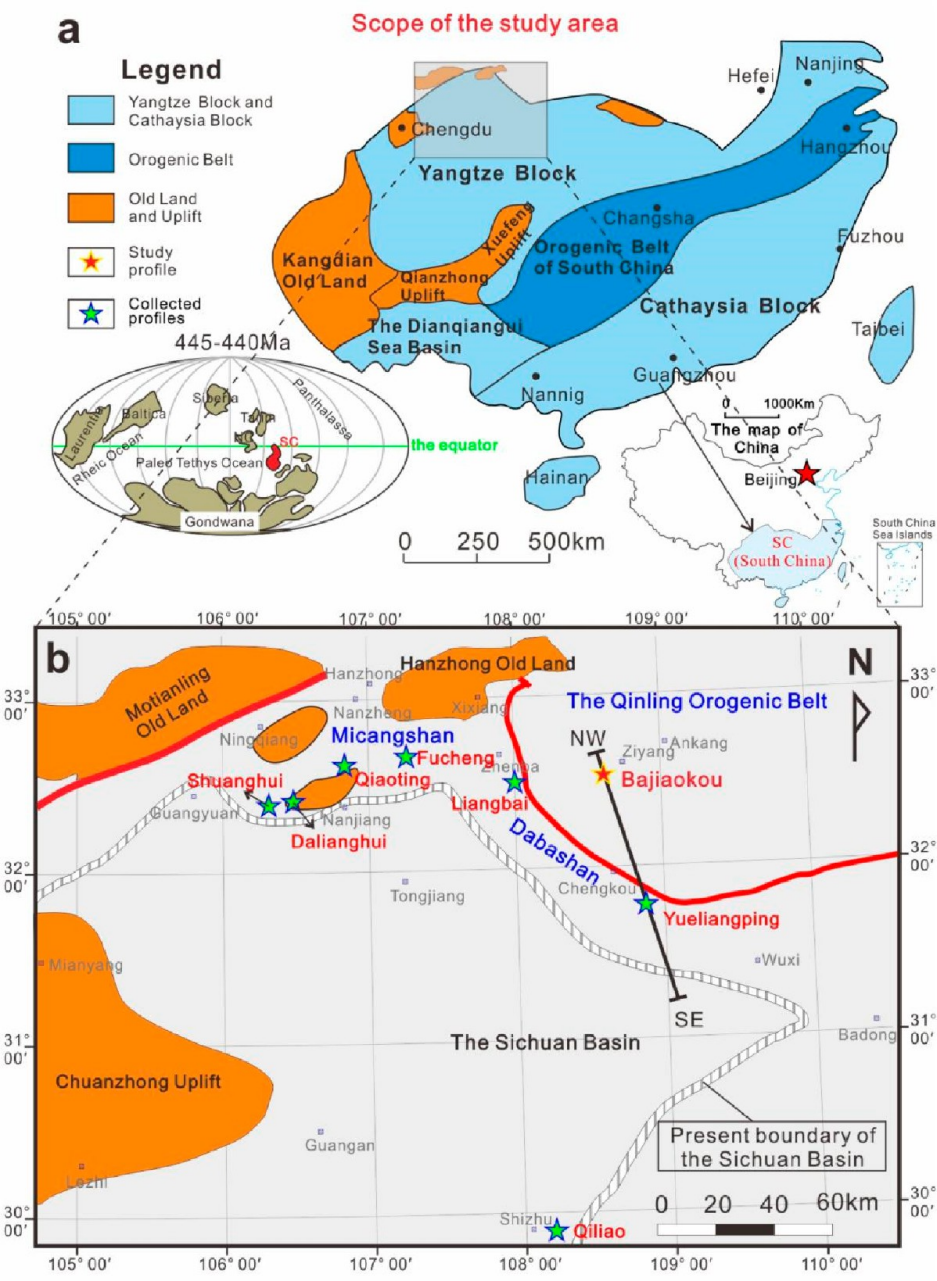
Paleogeographic location of the study area. (a) Location of the Yangtze block in the Late Ordovician. The illustration at the lower left corner shows the relative position of the Yangtze block on a reconstructed global paleogeographic map of the Late Ordovician. The diagram in the lower right corner indicates the geographical location of South China (according to the official Web site of the Ministry of natural resources of China). (b) The tectonic framework in the northern area of the Sichuan Basin and the southern area of the Qinling Orogenic Belt in the Late Ordovician and the location of the Yueliangping and Bajiaokou profiles.

During the Middle Paleozoic, South China constituted a distinct tectonic plate; however, it remained connected to the margins of Gondwana from the Late Ordovician to the Early Silurian (Figure 1a).^34,35^ The South China block at this time consisted of the Yangtze block in the northwest and the Cathaysia block in the southeast (Figure 1a).^36^ The compression of the Cathaysia block against the Yangtze block, along with the ongoing intracontinental orogeny, resulted in the isolation of most areas of the Yangtze block from the open sea.^36^ The Upper Yangtze Platform was encompassed by a series of uplifts and old lands, such as the Chuanzhong, Qianzhong, Xuefeng Uplift, and Kangdian Old Land, forming a partially enclosed bay that opened northwards during the Late Ordovician (Figure 1b).^37^

**Figure 2:**
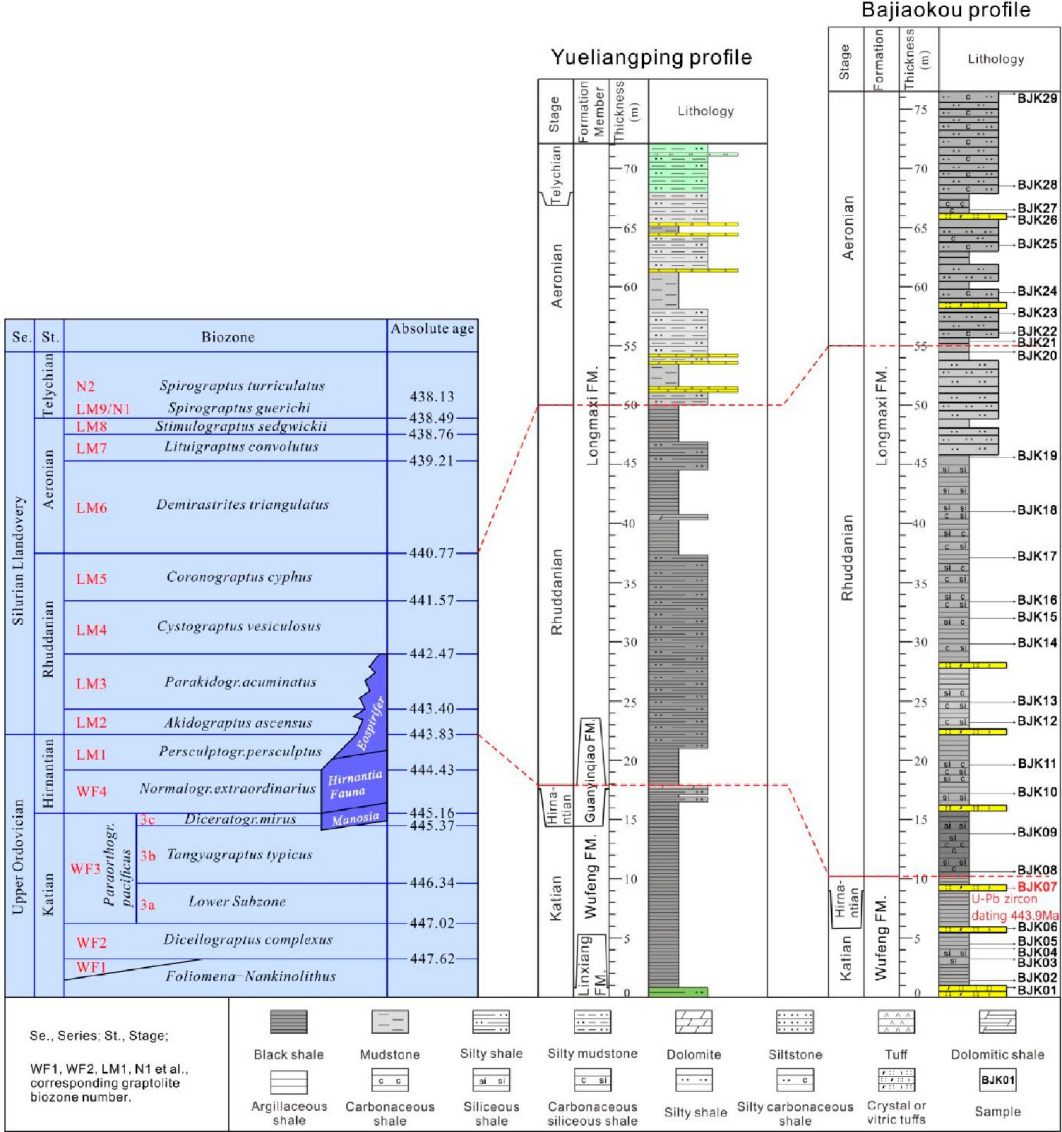
Stratigraphic correlation of the Bajiaokou profile of the South Qinling with the Yueliangping profile of the Sichuan Basin.

This paper mainly took the Bajiaokou profile located at the southern end of the Qinling orogenic belt as the research object. The Bajiaokou profile is exposed to the Bajiaokou Formation and the Maliushuwan Formation from bottom to top in sequence, corresponding to the Wufeng Formation and the lower part of the Longmaxi Formation in the Sichuan Basin of the Yangtze Platform in terms of the stratigraphic age. In order to facilitate a comparison of the profiles, the Wufeng–Longmaxi formations will be used for the following discussions in this paper. The stratigraphic development characteristics of the Bajiaokou profile were shown in Figure 2. In the Bajiaokou profile, there was no outcrop at the bottom of the Wufeng Formation and no outcrop at the top of the Longmaxi Formation, but the boundary between Wufeng–Longmaxi formations was quite clear. The LA-ICP-MS zircon U–Pb age measured from BJK07, a tuff sample collected near the top of the Wufeng Formation, is 443.9 ± 0.92Ma,^37^ which is basically consistent with the boundary age of 443.8 ± 1.5 Ma during the Ordovician and Silurian in the latest international chronostratigraphic table. The lithology of the Wufeng Formation in the Bajiaokou profile was dominated by argillaceous and siliceous shale, with visible bands of crystal and vitric tuffs, which were integrated totally with the overlying Longmaxi Formation (Figure 2). The lithology of the lower part of the Longmaxi Formation was dominated by the argillaceous and siliceous shale, with partially visible bands of the crystal and vitric tuffs (Figure 2). The upper part was dominated by the silty shale, with a gradually increasing content of the silty upward and without the exposure at the top (Figure 2).

## 3 Sample Analytical Methods and Data Analysis

### 3.1 Analytical Methods

Twenty-nine samples were selected from this profile, containing 3 tuff samples and 4 shale samples of Wufeng Formation (samples BJK-01 to BJK-07) and 1 tuff sample and 21 shale samples of Longmaxi Formation (samples BJK-08 to BJK-29). A total of 7 samples of Wufeng Formation and 22 samples of Longmaxi Formation were collected at intervals of 2.0–3.0 m (Figure 2).

The major element contents were analyzed using the alkali melting glass flake method at Nanjing University, Nanjing, China. The samples were ground to less than 200 mesh using a planetary crusher and baked for 24 h at 105 °C.^38^ Subsequently, an ARL-9800 X-ray fluorescence spectrometer (XRF) from the ARL Company in Switzerland was used for analysis, with an analytical error of less than 5.0% for the element concentrations. The specific sample handling process and testing method are detailed in previous studies.^38^

The trace element analysis was carried out at the Institute of Tibetan Plateau Research, Chinese Academy of Sciences, using the acid solution method and a Thermo Fisher X Series ICP-MS. The detection limits for the trace elements ranged from 0.*n* × 10^–12^ to *n* × 10^–12^ (*n* = 1–9), with an analytical error typically better than ±0.5%. The specific sample handling process and testing method are detailed in previous studies.^39^

The total organic carbon (TOC) content was analyzed at the Beijing Research Institute of Uranium Geology. The washed sample was ground to 80.0 mesh using an agate mortar, and then 100.0 mg of the sample was dissolved in 5.0% dilute hydrochloric acid until no bubbles were produced. After soaking for 24 h to remove carbonate and other inorganic carbon, analysis was conducted using a German Eltra CS580A carbon and sulfur analyzer. Based on repeated analysis of standard samples, the accuracy of TOC content analysis is better than ±0.5%.^39^ Contents of TOC and major-element oxides of samples in the Bajiaokou profile are given in Supporting Information Table 1.

### 3.2 Data Analysis

The variability of major elements is examined both as absolute concentrations and after normalization to Al. The presence of aluminum is confined to the detrital aluminosilicate component in near-shore environments,^28^ thus allowing for the investigation of elemental concentrations from nonaluminosilicate sources (e.g., biogenic dilution of silica and carbonates). Element concentrations are compared to that of average shale,^40^ and element enrichment factors (EF_element_) are determined using the formula:^41,42^
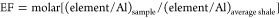
1The average major element concentrations and EFs are given in Table 1.

**Table 1:** Average Aluminum (Al) Normalized Concentrations of All Elements in the Bajiaokou Profile^a^

	Average shale		Wufeng FM (*n* = 6)			Longmaxi FM (*n* = 28)		
Oxide/element	Abundance	/Al	Abundance	/Al	EF	Abundance	/Al	EF
TOC (wt %)	0.20	0.01	0.22	0.01	1.15	0.64	0.04	3.00
SiO_2_ (wt %)	58.90	3.53	68.00	4.21	1.25	63.36	3.66	1.07
TiO_2_ (wt %)	0.78	0.05	1.06	0.07	1.50	1.07	0.06	1.33
Al_2_O_3_ (wt %)	16.70	1.00	16.14	1.00	1.00	17.33	1.00	1.00
TFe_2_O_3_ (wt %)	6.92	0.41	4.63	0.29	0.68	5.96	0.34	0.84
MnO (wt %)	0.09	0.01	0.03	0.00	0.34	0.04	0.00	0.43
MgO (wt %)	2.60	0.16	1.71	0.11	0.68	2.26	0.13	0.84
CaO (wt %)	2.20	0.13	0.32	0.02	0.17	0.28	0.02	0.13
Na_2_O (wt %)	1.60	0.10	0.48	0.03	0.32	0.26	0.02	0.17
K_2_O (wt %)	3.60	0.22	3.68	0.23	1.07	4.05	0.23	1.08
P_2_O_5_ (wt %)	0.16	0.01	0.12	0.01	0.86	0.17	0.01	1.02
V (μg/g)	130.00	7.78	143.2	8.9	1.2	175.0	10.1	1.3
Mo (μg/g)	2.60	0.16	2.3	0.1	1.0	4.3	0.3	1.6
Zr (μg/g)	160.00	9.58	208.5	12.9	1.4	229.8	13.3	1.4
Ba (μg/g)	580.00	34.73	2882.5	178.6	5.2	3422.0	197.5	5.7
U (μg/g)	3.70	0.22	3.2	0.2	0.9	4.0	0.2	1.1

a Average shale composition is from Wedepohl (1971).^36^

The concentrations of Si derived from biogenic sources (excess Si or Si_XS_) were calculated using the following formula:^42^
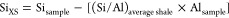
2where the value of (Si/Al)_average shale_ is 3.11. Si_XS_ is mainly attributed to biogenic silica and can be used as a productivity proxy.^43^ The Si_XS_ values are given in Supporting Information Table 2.

Figure 9 shows a plot of Co[EF] and Mn[EF] from studied samples normalized using post-Archean Australian shale (PAAS) data.^41,44^ Specifically, the enrichment factor of element X is determined using the formula:^41^
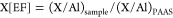
3where X and Al represent the weight concentrations of elements X and Al, respectively. PAAS represents the composition of the post-Archean Australian shale.^44^ See Supporting Information Table 3 for sample test information (please refer to Supporting Information Tables 4–7 for sample test results).

## 4 Results

### 4.1 TOC Contents

The analytical results of TOC contents and major element concentrations for the Bajiaokou profile are listed in Supporting Information Table 1. The shale samples of Wufeng Formation display marked reduction of organic matter, the TOC content of which varies from 0.15 to 0.28 wt % and averages 0.22 wt % (Supporting Information Table 1). Subsequently, the TOC content of Longmaxi Formation increased to 1.02 wt % in the carbonaceous shale (Supporting Information Table 1). The TOC content of Longmaxi Formation fluctuated greatly, ranging from 0.06 wt % to 2.08 wt %, with an average of 0.64 wt %.

### 4.2 Major Element Geochemistry

The results of major element analyses are presented in Supporting Information Table 1. SiO_2_ is the dominant constituent of the studied samples, averaging 64.11 wt % and varying from 55.73 to 78.59 wt %. Al_2_O_3_ is the second most abundant component with values ranging from 9.92 to 19.52 wt % (average 17.14 wt %). The sum of the two major components ranges from 73.55 wt % to 88.51 wt %, with an average of 81.25 wt %.

**Figure 3:**
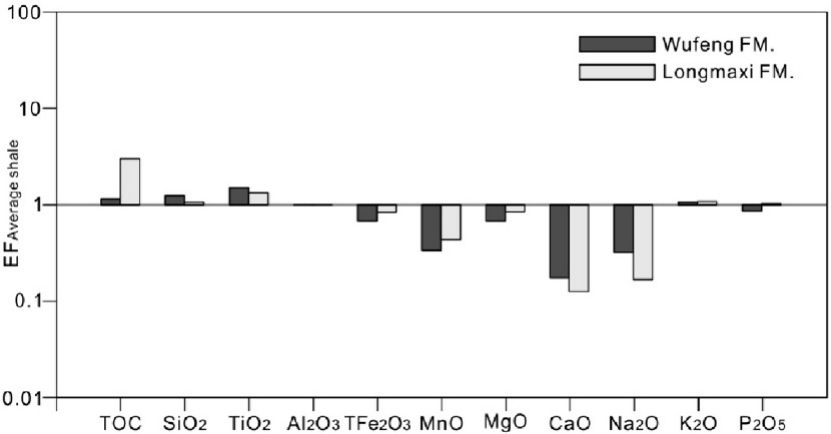
Enrichment factors of major elements and the TOC content from the Wufeng–Longmaxi formations in the Bajiaokou profile are shown with a horizontal line drawn at an EF of 1 to indicate element enrichment or depletion relative to average shale.

Figure 3 illustrates that the Wufeng Formation of the Bajiaokou profile exhibits high concentrations of SiO_2_, TiO_2_, and K_2_O, while the Longmaxi Formation is characterized by elevated levels of SiO_2_, TiO_2_, K_2_O, and P_2_O_5_. The increased K_2_O content may indicate a higher presence of Illite. The presence of SiO_2_, Al_2_O_3_, and TiO_2_ is primarily associated with quartz and clay minerals.

**Figure 4:**
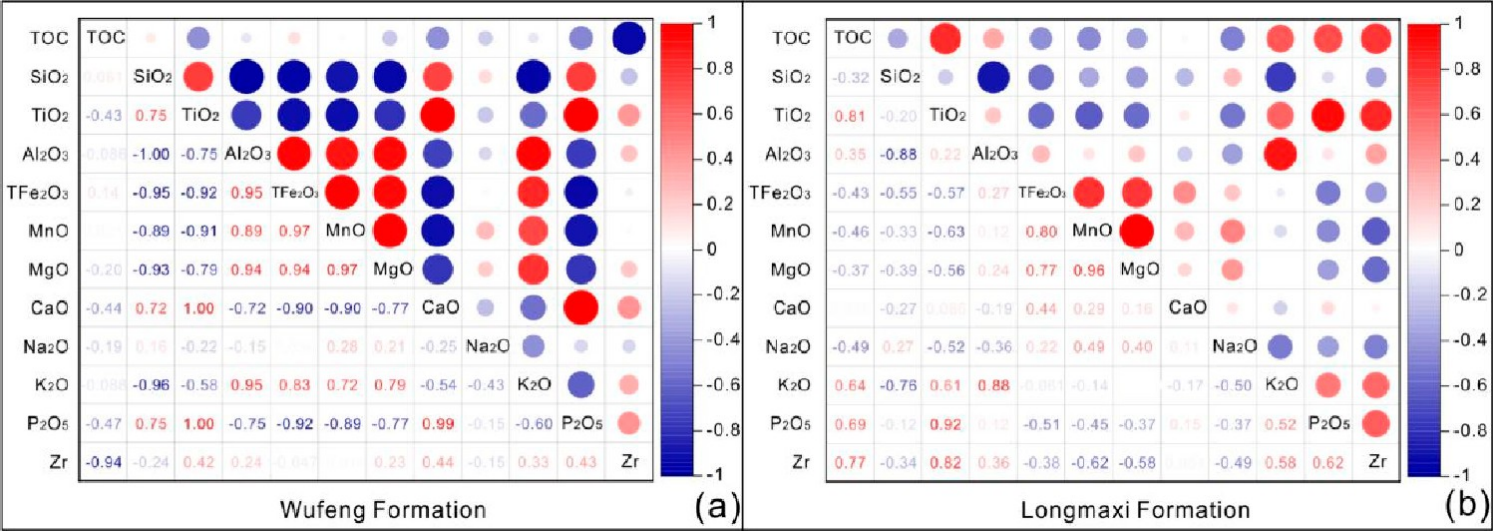
Heat map of Pearson correlation analysis between TOC, Zr, and major elements in shale of Wufeng Formation (a) and Longmaxi Formation (b) in the Bajiaokou profile.

In marine sediments, TiO_2_ is a stable component in diagenetic processes with an enrichment factor (EF) greater than 1.0 (Figure 3). By analyzing the correlation of elements, it was found that the correlation coefficient between TiO_2_ and Al_2_O_3_ in the Wufeng Formation was −0.75, and that between TiO_2_ and Al_2_O_3_ in the Longmaxi Formation was 0.23. Meanwhile, it showed that TiO_2_ had a good positive correlation with Zr, suggesting that the Al in the Bajiaokou profile came from the common siliceous detritus source region, while the Ti element may have different sources, such as autogenetic enrichment or pyroclastic material (Figure 4).^42^ Under low-energy hydrodynamic depositional conditions, Ti is mainly associated with terrestrial debris and fine-grained sediments, which is also the reason for the lower Ti/Al ratios in the Wufeng–Longmaxi formation shale (Table 1).^11,28^

### 4.3 Chemical Index of Alteration (CIA)

In the study of fine-grained sediments, CIA is often used to reconstruct the paleoclimate of the sediments.^9,45,46^ The CIA can be calculated by molar [(Al_2_O_3_)/(Al_2_O_3_ + CaO* + Na_2_O + K_2_O)] × 100, where CaO* only represents the content of CaO in silicate minerals.^45^ Sedimentary rocks usually contain some carbonate minerals and apatite; therefore, the CaO content of the sample must be corrected. In this study, the obtained P_2_O_5_ data were used for preliminary calibration of CaO according to (CaO* = mole CaO – mole P_2_O_5_ × 10/3).^46^ When the residual molar number is less than Na_2_O, the CaO value is used as the CaO* value; otherwise, the Na_2_O value is used as the CaO* value.^46^

**Figure 5:**
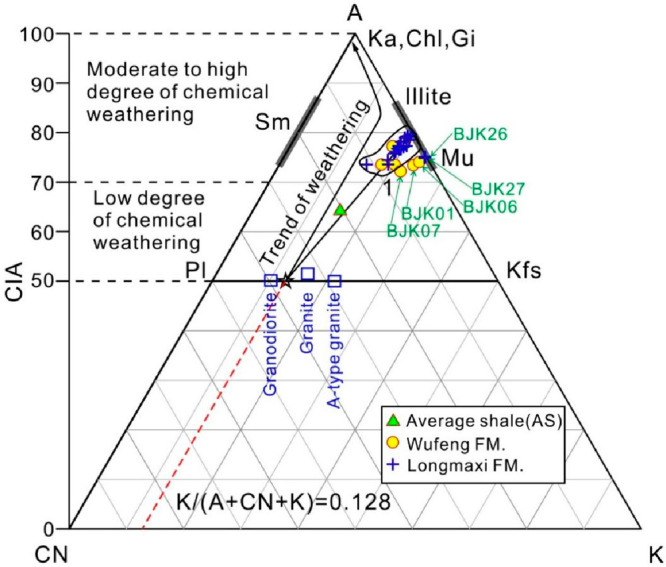
A–CN–K ternary diagram. The changes of chemical composition and corresponding CIA of the shales of Wufeng–Longmaxi formations in the Bajiaokou profile are revealed. The ideal weathering trend line is represented by a solid line with an arrow parallel to the A–CN boundary line. The ideal weathering trend line of the bedrock in the figure is used to compare the degree of weathering deviation of the test sample. K-feldspar (Kfs); Plagioclase (Pl); Smectite (Sm); Kaolinite (Ka); Chlorite (Chl); Gibbsite (Gi); and Muscovite (Mu).

Figure 5 shows the ternary diagram of A–CN–K (Al_2_O_3_–CaO* + Na_2_O–K_2_O), and the value of CIA can reflect the degree of chemical weathering experienced by the sediment. Typically, high CIA values (80–100) represent sediments that have undergone strong chemical weathering, while areas with low CIA values (50–70) represent initial weathered sediments.^9,45^ Theoretically, progressive weathering products usually produce a series of CIA values, which are distributed along a straight line (ideal weathering trend) parallel to the A–CN subline in Figure 5.^47^ The CIA values calculated in this study are shown in Supporting Information Table 2. The CIA values of the samples of the Wufeng Formation ranged from 72.2 to 78.4 (average 74.63), indicating a moderate degree of chemical weathering. The CIA values of the samples from Longmaxi Formation ranged from 73.56 to 79.59 (average 76.72), indicating a moderate degree of chemical weathering (Figure 5).

### 4.4 Paleoenvironment Proxies

**Figure 6:**
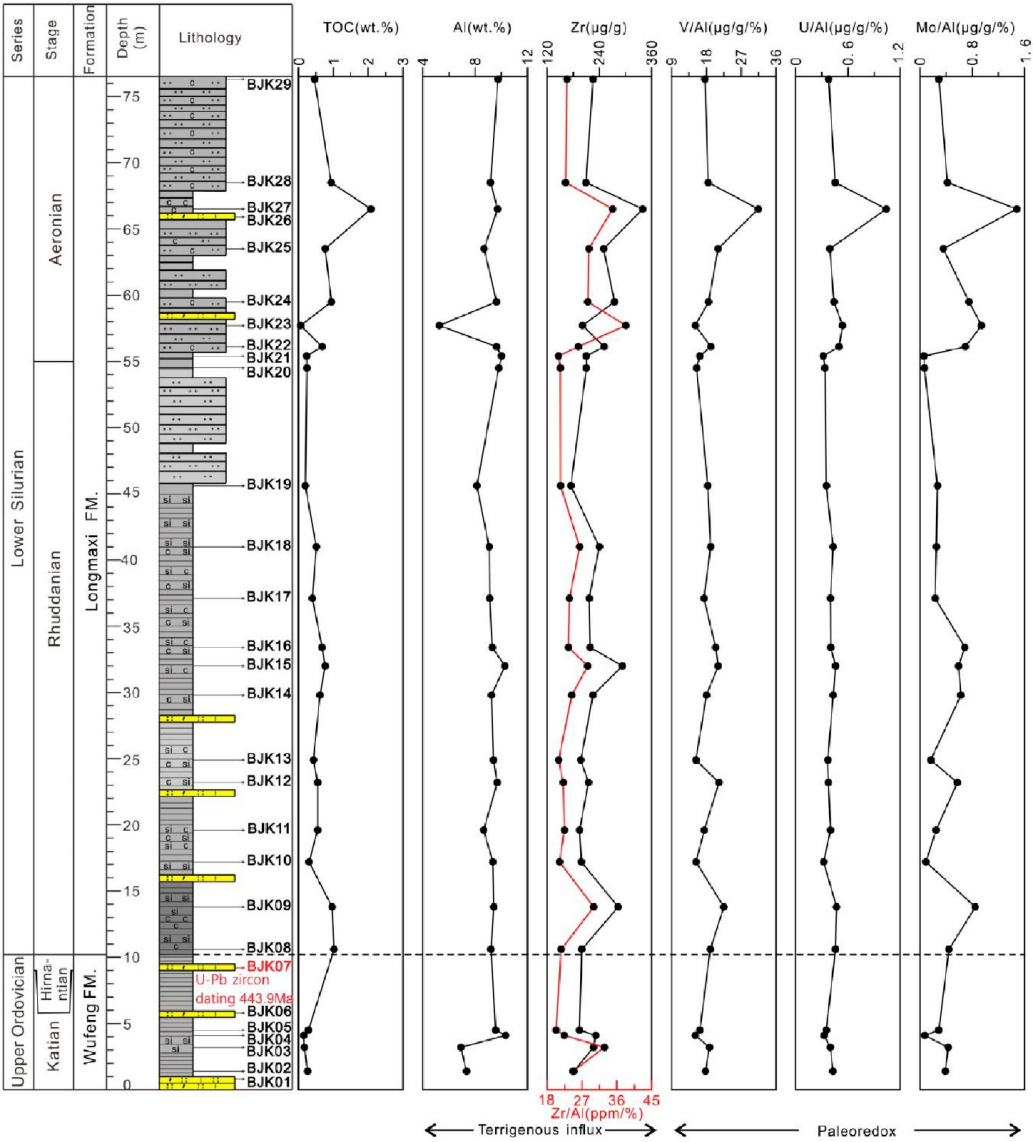
Vertical variation trend of shale terrigenous clastic flux and redox condition indices from the Wufeng Formation to the Longmaxi Formation of the Bajiaokou profile. Legend is shown in Figure 2.

The elements Al and Zr in sedimentary rocks are rarely affected by weathering during sedimentary diagenesis and are often used as indicators to determine the flux of terrestrial debris in sediments.^41^ In the Bajiaokou profile, detrital proxies Al and Zr exhibit different variation patterns (Figure 6). The Al content of the shale in the Wufeng Formation showed a trend of increasing from the bottom to the top (Figure 6), with a content of 6.91–10.33 wt % (average 8.54 wt %). The Zr content showed a trend of first increasing and then decreasing, with a content of 181.30–231.60 μg/g (average 208.45 μg/g). The overall fluctuation of Al content in the Longmaxi Formation shale was not obvious (Figure 6), with only a low value of 5.25 wt % observed in the sample BJK23, which was obtained in the upper part. The other samples, in which the Al content ranged from 8.13 to 10.28 wt % (average 9.37 wt %), had significant fluctuations in Zr content, ranging from 174.60 to 339.30 μg/g (average 229.77 μg/g).

Trace elements V (vanadium), U (uranium), and Mo (molybdenum) will change their elemental valence when the external redox conditions change. These elements are considered to be redox sensitive elements. Their accumulation and loss in sediments are mainly affected by environmental redox conditions.^41^ These three elements have a similar trend with TOC (Figure 6). The Wufeng–Longmaxi formations show little or no enrichment of V, U, and Mo (Figure 6).

**Figure 7:**
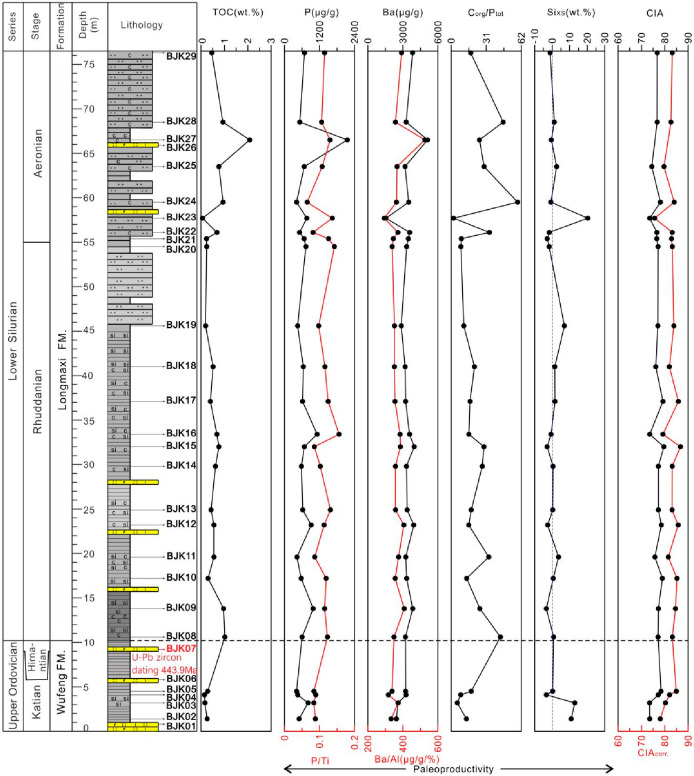
Vertical variation trend of shale CIA and primary productivity indices in the Wufeng Formation to Longmaxi Formation in the Bajiaokou profile.

Phosphorus/titanium (P/Ti) and barium/aluminum (Ba/Al) in sedimentary rocks are two commonly used geochemical parameters to judge the productivity of organic matter.^48,49^ P (phosphorus) is generally considered an important nutrient for plankton in the ocean and is one of the most basic limiting elements in the marine environment.^48−50^ In addition, P is still the main component of skeletal material in the growth process of microorganisms and animals.^39^ P plays a crucial role in biological metabolism. Therefore, P is usually used to infer paleoproductivity.^51,52^ Usually in organic-rich shales, organic matter and authigenic minerals dilute the content of absolute phosphorus in terrigenous clastic materials.^53^ This dilution can be eliminated using Ti and Al elements representing terrigenous clastic material, and paleoproductivity can be assessed using P/Ti or P/Al ratios.^28,51,54^ Similar to phosphorus, Ba (barium) plays an important role in the metabolism of many marine organisms, so barium in shale is often used to assess marine primary productivity during sedimentation.^55^ The calculation results of P/Ti and Ba/Al ratios are shown in Supporting Information Table 2. Correlation analysis of P/Ti-TOC and Ba/Al-TOC of the samples of the Bajiaokou profile showed that no meaningful correlation was obtained (Figure 7). In order to evaluate whether phosphorus in sediments has undergone recycling, that is, to understand redox-dependent phosphorus burial and recycling under marine conditions, the *C*_org_/*P*_tot_ ratio can be used.^56,57^ The *C*_org_/*P*_tot_ ratios of the Wufeng Formation decreased first and then increased, ranging from 5.36 to 17.67, with an average of 11.2 (Figure 7). The *C*_org_/*P*_tot_ ratios of the Longmaxi Formation change frequently, ranging from 2.05 to 58.7, with an average of 23.71 (Figure 7).

## 5 Discussion

### 5.1 Climate Change and Denudation during the Ordovician–Silurian Transition

The composition of most siliceous detrital sedimentary rocks is closely related to the formation of weathering profiles but not closely related to the composition of underlying bedrock.^58^ In the ternary diagram of A–CN–K, due to the metasomatism of K ions on some minerals, the actual weathering trend of the sample deviates from the ideal weathering trend line of the parent rock. The intersection point between the actual weathering trend line fitted to the sample data points and the feldspar line can reflect the proportion of feldspar in the unweathered parent rock. The intersection point between the actual weathering trend line and the feldspar line can be used to infer the ideal weathering trend line. The ideal weathering trend line is extended in reverse and intersects with the CN–K boundary line, with an “*m*” value of K/(A + CN + K). The K_2_O content of the sample was corrected by “*m*” value; i.e., K_2_O_corr._ = [*m* × A + *m* × (C + N)]/(1 – *m*), and then the corrected K_2_O_corr._ was used for the following calculation, i.e., CIA_corr._ = molar [(Al_2_O_3_)/(Al_2_O_3_ + CaO* + Na_2_O + K_2_O_corr._)] × 100 to obtain CIA_corr_.

According to statistics, the CIA values of fine-grained argillaceous rocks range from 85 to 100, which usually indicates that the sedimentary period is in hot and humid tropical climates; CIA values range from 70 to 85, indicating a warm and humid climate during the deposition period; and CIA values range from 50 to 70, indicating a cold and arid climate during the deposition period.^9,45,58^

The shales of Wufeng–Longmaxi formations in the Bajiaokou profile were mainly concentrated on the area of medium to high degree of chemical weathering in the A–CN–K ternary diagram (Figure 5). It was worth mentioning that the four tuff samples (BJK01, BJK06, BJK07, BJK26) and one shale sample (BJK27) with TOC content of 2.08 wt % collected from the Bajiaokou profile showed an obvious dispersion compared with the other shales in the A–CN–K ternary diagram, which may indicate differences in the source areas between these five samples and the other shales.

The CIA_corr._ values of Wufeng Formation shales increased from bottom to top, ranging from 75 to 85 (Figure 7), indicating that the climate in the sediment source area was warm and humid. The CIA_corr._ values of Longmaxi Formation shales fluctuated slightly, most of them ranging from 75 to 85, with 5 samples ranging from 85 to 87 (Figure 7), indicating that the climate of the sediment source area was basically the same as that of the Wufeng Formations, belonging to a warm and humid climate. With the increase of the fluctuation, it could lead to a tropical humid and hot climate. Those values are basically consistent with the data reported by Yan et al.^9^ (2010), which reveals that the CIA_corr._ values of Wufeng–Longmaxi formations are mainly clustered between 70 and 85. This similarity may indicate that during the transition period from Late Ordovician to Early Silurian the climate of the southern margin of the Qinling orogenic belt was basically consistent with that of the central and eastern parts of the Yangtze platform.

### 5.2 Terrigenous Flux

In sedimentary rocks, Al (aluminum) generally exists in aluminosilicate clay minerals.^41^ Zr (zirconium) in sedimentary rocks is generally found in silt-sized minerals or clay minerals such as zircon.^59^ Therefore, the Zr/Al ratio is often used as a proxy for the coarse-grained composition of the sediment.^46,59^ The Al content, Zr content, and Zr/Al ratio of Wufeng Formation shale in the Bajiaokou profile showed opposite trends. The Al content decreased first and then increased, while the Zr content and Zr/Al ratio increased first and then decreased (Figure 6), indicating that the flux of aluminosilicate clay minerals in the terrigenous clastic fluxes of the Wufeng Formation decreased first and then increased. The flux of silt grade granular minerals increased first and then decreased. The correlation between Al and Zr is only moderately positive, indicating that the increased flux of silt grade particles may not necessarily be terrigenous clasts but may also be from other sources. It is inferred from the large number of tuff interlayers developed in the Wufeng Formation that the flux of silt grade granular minerals may be a source of pyroclastic. The content of Al and Zr and the ratio of Zr/Al in the Longmaxi Formation shale showed the same variation trend as a whole (Figure 6). The flux of terrigenous debris in the Longmaxi Formation presents very slight variations, but the amplitudes of changes in individual samples vary greatly. The Zr/Al ratio shows the opposite change only in sample BJK23. Four samples, BJK09, BJK15, BJK22, and BJK27, showed a significant increase in Zr content and Zr/Al ratio compared to the Al content. The correlation between Al and Zr is only moderately positive, indicating that the clastic flux of the four samples increased significantly during the deposition, and the composition of the four samples was mainly silt grade granular minerals; however, the source of the detrital materials may not be all terrigenous detritus but also may be pyroclastic. Sample BJK23 shows an increase in the ratio of Zr/Al and a decrease in Al and Zr contents, indicating that there is a significant decrease in the content of aluminosilicate clay minerals in the terrestrial debris flux during sedimentation, while the content of fine-grained particle minerals increases significantly. The TOC of the Wufeng Formation shale is not significantly correlated with Al but shows a significant negative correlation with Zr, suggesting that the TOC is diluted by the flux of the fine-grained mineral debris, suggesting that it may be diluted by a large amount of pyroclasts (Figure 6). The TOC of the Longmaxi Formation shows a certain positive correlation with Al and a significant positive correlation with Zr. Since the TOC content in the Bajiaokou profile is generally lower, the positive effect of this debris flux on the TOC may not be significant. It is speculated that the substances in the debris flux may have a certain promoting effect on the production of organic matter, but the content of organic matter in the sediments may also be affected by other factors, such as redox conditions. The details will be analyzed and discussed in the following parts.

### 5.3 Paleoredox Conditions

In marine sedimentary rocks, V (vanadium), U (uranium), and Mo (molybdenum) elements are relatively rich, and these elements have variable valence states and selectively accumulate in water bodies and sediments according to the changes of water redox conditions. Therefore, the elements V, U, and Mo are used to study the paleoredox conditions of marine sedimentary rocks.^60^

Compared to oxic sediments, V is preferentially enriched in anoxic sediments.^41^ Two reduction reactions are beneficial for removing V from the water bodies into the sediments: under nonsulfurized anoxic conditions and under sulfurized euxinic conditions.^61^ Under nonsulfurized anoxic conditions, the abundance of V is usually closely related to TOC concentration, as humic acid and fulvic acid accelerate the reduction of V (V) to V (IV). On the contrary, the reduction of V(IV) to V(III) under sulfurized conditions does not depend on organic reactions.^54^

U generally exists in the form of solubility of uranyl carbonate complexes [UO_2_(CO_3_)4-3] in oxic to dysoxic seawater, but under under reducing conditions, it can be reduced to U (IV) precipitation into sediments.^28,62^ This is because, in organic rich sediments, bacterial sulfate reduction (BSR) can to some extent reduce U (VI) to U (IV), and the strength of sulfate reduction activity is closely related to the abundance of organic matter. Therefore, under nonsulfurized anoxic conditions, the content of TOC in sediment controls the degree of U enrichment.^30,41^

Mo and U are usually enriched in sediments under anoxic and euxinic water body conditions.^11,30^ However, the geochemical processes of the enrichment of these two elements are different. In marine sediments, the enrichment of U depends on the absorption of authigenic U, a process that begins at the Fe(II)–Fe(III) redox boundary (i.e., dysoxic conditions). Euxinic conditions in the presence of hydrogen sulfide are the key factors leading to authigenic Mo enrichment in marine sediments.^63^

When the paleoredox conditions in the study profile were evaluated using V/Al, U/Al, and Mo/Al ratios, it was observed that relative changes in redox conditions along the vertical direction can be discerned within a single profile; however, the qualitative assessment of the degree of redox conditions was not feasible. Hence, a range of commonly used element ratios such as U/Th, V/Cr, and V/Sc were introduced as indicators for discriminating redox conditions in order to comprehensively assess the paleoredox conditions of the study profile. As mentioned above, element U had a variable valence state, while element Th was not affected by redox conditions. Therefore, it was considered that the U/Th ratio could be used as an indicator to distinguish redox conditions. Generally, the value of U/Th, less than 0.75, indicates oxidation conditions. The U/Th value of 0.75–1.25 indicates dysoxic conditions, and the U/Th value greater than 1.25 indicates anoxic conditions.^64^ The enrichment level of element V could be corrected by the abundance of the Sc element, with V/Sc values high in anoxic conditions and low in oxic conditions.^65^ In the euxinic conditions, the elements V, U, and Mo are prone to react with HS^–^ to generate the corresponding sulfides and then to accumulate, while the element Cr reacts slowly with HS^–^, resulting in a lower enrichment rate than V, U, and Mo. Moreover, under anoxic conditions, element V is more likely to be enriched than element Cr in sedimentary rocks containing organic matter. Accordingly, it is proposed that the ratio of V/Cr can be used as an indicator to distinguish between redox conditions. The V/Cr value less than 2 indicates oxic conditions. The V/Cr value ranging from 2 to 4.5 indicates dysoxic conditions. The V/Cr value greater than 4.5 indicates anoxic conditions.^64^

**Figure 8:**
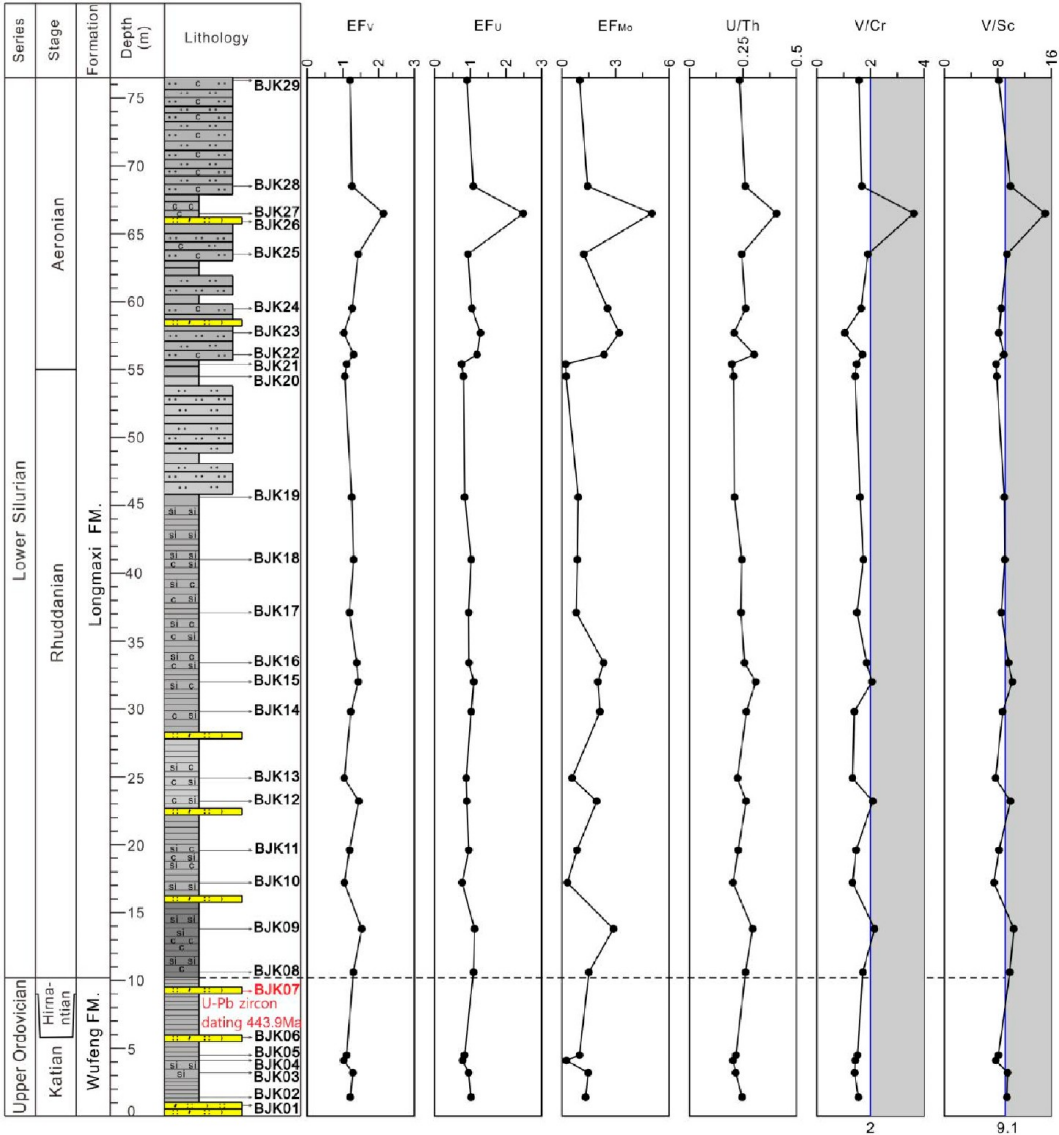
Vertical variation trend of U/Th, V/Cr, and V/Sc indices from the Wufeng Formation to Longmaxi Formation in the Bajiaokou profile.

The shales of Wufeng–Longmaxi formations from the Bajiaokou profile show basically the same variation trend of V/Al, U/Al, and Mo/Al. Only in the middle and upper part of Longmaxi Formation is the variation trend of V/Al opposite to that of U/Al and Mo/Al, showing a similar or slight enrichment compared with the average shale as a whole (Figure 6). The EF_V_ and EF_U_ of shale in Wufeng–Longmaxi formations in the Bajiaokou profile are between 0 and 3 as a whole, while the EF_Mo_ fluctuates between 0 and 6. Its change trend is basically consistent with the ratio of U/Th, V/Cr, and V/Sc (Figure 8). The U/Th ratio reflects that the whole profile is under oxic conditions. From the V/Cr and V/Sc ratios, it reflects that the whole profile fluctuates slightly near the critical value of oxic conditions and dysoxic conditions. From the locally developed black and gray-black shales of Wufeng–Longmaxi formations, it is speculated that the oxic–dysoxic conditions reflected by the V/Cr and V/SC ratios may be more consistent with the actual situation. The TOC contents of shales in Wufeng–Longmaxi formations have a similar variation trend with V/Al, U/Al, and Mo/Al (Figure 8), indicating that the TOC contents are controlled by the bottom water conditions of overall oxic–dysoxic to some extent.

### 5.4 Paleoproductivity

In the shale of Wufeng–Longmaxi formations in the Bajiaokou profile, the variation trends of P and P/Ti, Ba, and Ba/Al are basically the same (Figure 7), indicating that components related to Ti and Al have no obvious dilution effect on the elements P and Ba. Although the Ti element may have nonterrigenous clastic sources, it has little effect on the P/Ti index. The negative values of Si_XS_ indicate that the Si element is mainly derived from the terrigenous clasts related to aluminosilicate.

The P/Ti ratio of the Wufeng Formation shale fluctuates slightly, with an average of 0.08 (Figure 7). The Si_XS_ values of the two samples at the bottom of the Wufeng Formation are 10.87 and 12.91 wt %, respectively, while the samples above have negative values (Figure 7). Negative Si_XS_ values indicate that there is no biological silicon or that the proportion of biological silicon is very small. The Wufeng Formation has a moderate to low level of primary productivity as a whole. The P/Ti ratio of shale in the Longmaxi Formation fluctuates in some samples, while the overall change is gentle, with an average value of 0.11, only four samples being less than 0.10. The overall Si_XS_ value of shale in the Longmaxi Formation is between 0 and 7 wt %, and only the sample BJK23 is 20.34 wt %. The Longmaxi Formation shows a moderate level of primary productivity as a whole. Compared with the Yueliangping profile in the northern margin of the Sichuan Basin, the Bajiaokou profile lacks siliceous radiolarians and other organisms as a whole in the seawater during the sedimentary period of the Wufeng–Longmaxi formations.^39^

### 5.5 Identifcation of Watermass Restriction Degree

**Figure 9:**
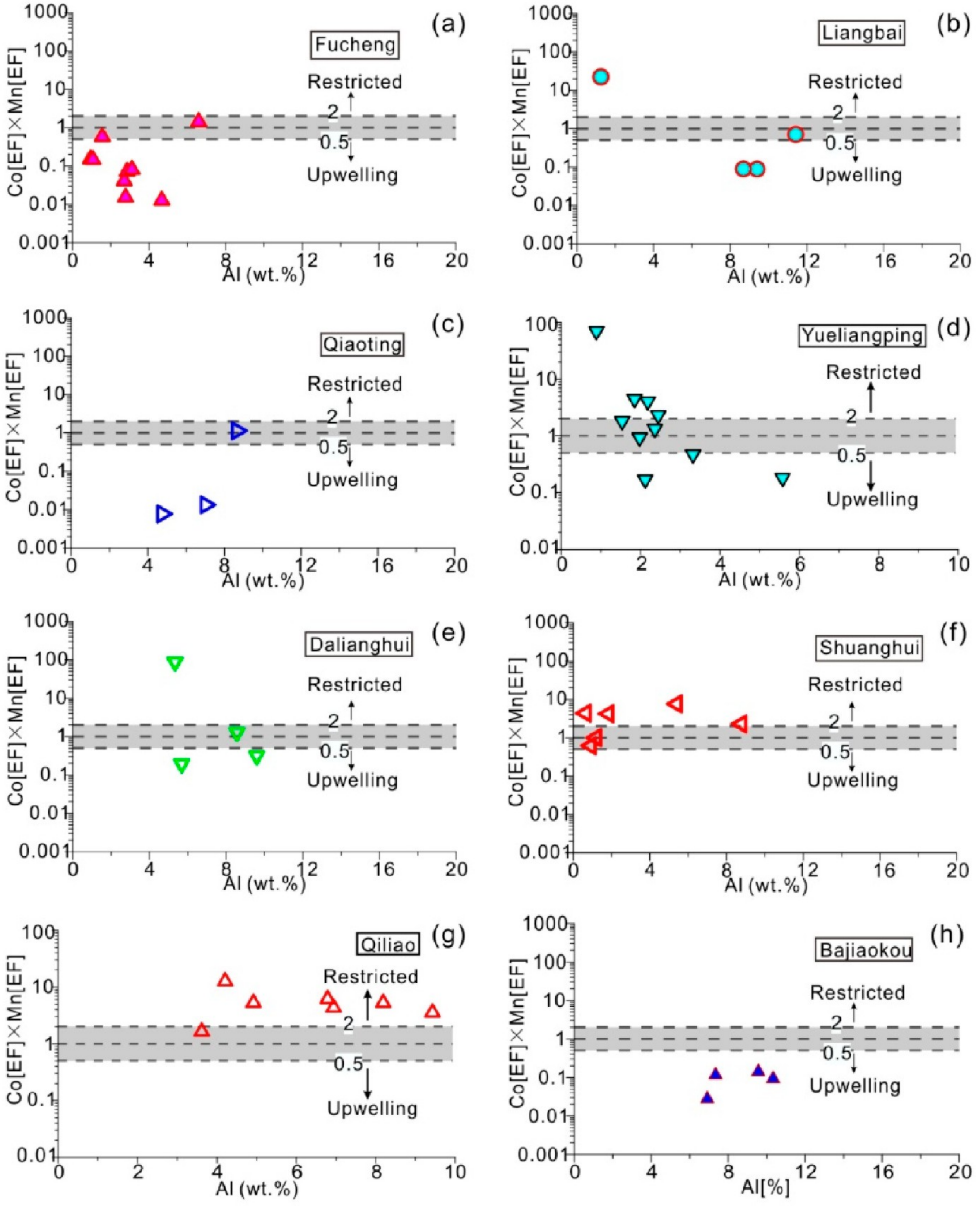
Co [EF] × Mn [EF] versus Al diagram of water mass restricted degree analysis in the Wufeng Formation. (a) Fucheng profile. (b) Liangbai profile. (c) Qiaoting profile. (d) Yueliangping profile. (e) Dalianghui profile. (f) Shuanghui profile. (g) Qiliao profile. (h) Bajiaokou profile.

Previous studies have proposed that, based on the unique geochemical properties of U and Mo in seawater, MO-TOC and U-MO covariant patterns in sediments can be used to determine the degree to which seawater is restricted.^29,66^ This research result has been widely used, especially in the study of confined anaerobic basins.^11,67,68^ The hydroxide particles of Mn and Fe can adsorb Mo in seawater, leading to a significant enrichment of Mo in sediments,^69^ which will result in that the Mo-TOC covariation model cannot effectively distinguish the degree of water restricted in the study of continental margin marine systems. Sweere et al.^33^ (2016) studied the migration path of metal elements Mn and Co into marine sediments in modern oceans and found that: (1) with the increase of water depth, reactive oxygen species in seawater continue to decrease, and dissolved Mn and Co in seawater also gradually decrease^70,71^ and (2) when the seawater was strongly reduced, Mn in the water was more soluble, and Co was more easily entered into the sediment.^41,72^ Based on the above research finding, Sweere et al.^33^ (2016) proposed that the Al–Co × Mn discriminant diagram could be used to distinguish whether the water body during the period of marine sediment deposition was in a restricted environment or in an upwelling area/open sea. The study of modern marine sediments by this method showed that the Al–Co × Mn discriminant diagram could effectively identify the sediments of modern typical continental margin upwelling zones (such as the Peruvian Margin) and typical restricted basins (such as the Black Sea). In Figure 9, the Al–Co[EF] × Mn[EF] discriminant diagram was used. The enrichment factors of Co and Mn in sediments were calculated in order to eliminate the contribution of clastic materials and the dilution effect on authigenic elements.

#### 5.5.1 Water Restricted Degree of Wufeng Formation

During the sedimentary period of the Wufeng–Longmaxi formations, a series of uplifts and old lands emerged in the western, eastern, and southern regions of the Sichuan Basin, which led to the formation of a semienclosed, limited shallow marine environment that was only connected to the vast sea area in the north.^73^

Previous studies on the stratigraphic framework and correlation analysis of the Wufeng Formation in the northern margin of the Sichuan Basin, conducted through field exposures and borehole profiles, have revealed that the Micangshan region, situated north of the “Chuanzhong Uplift”, extends from south to north toward the periphery of the Hanzhong Old Land.^37^ During the sedimentation of the Wufeng Formation, the “Xixiang Rise” at the forefront of Micangshan did not exert any influence on the Dalianghui and Qiaoting areas. In the Dalianghui area, there was a relatively low-lying terrain, where thick carbonaceous shale intercalated with siliceous rock was deposited. The Dabashan area, located to the east of Hanzhong Old Land and south of Chengkou fault with its northern boundary adjacent to the open sea, is significantly distant from the influence range of “Xixiang Rise”. It is characterized by low terrain and a deep water depth. Across all profiles, the Wufeng Formation exhibits development with siliceous shale and local occurrences of siliceous rock.

**Figure 10:**
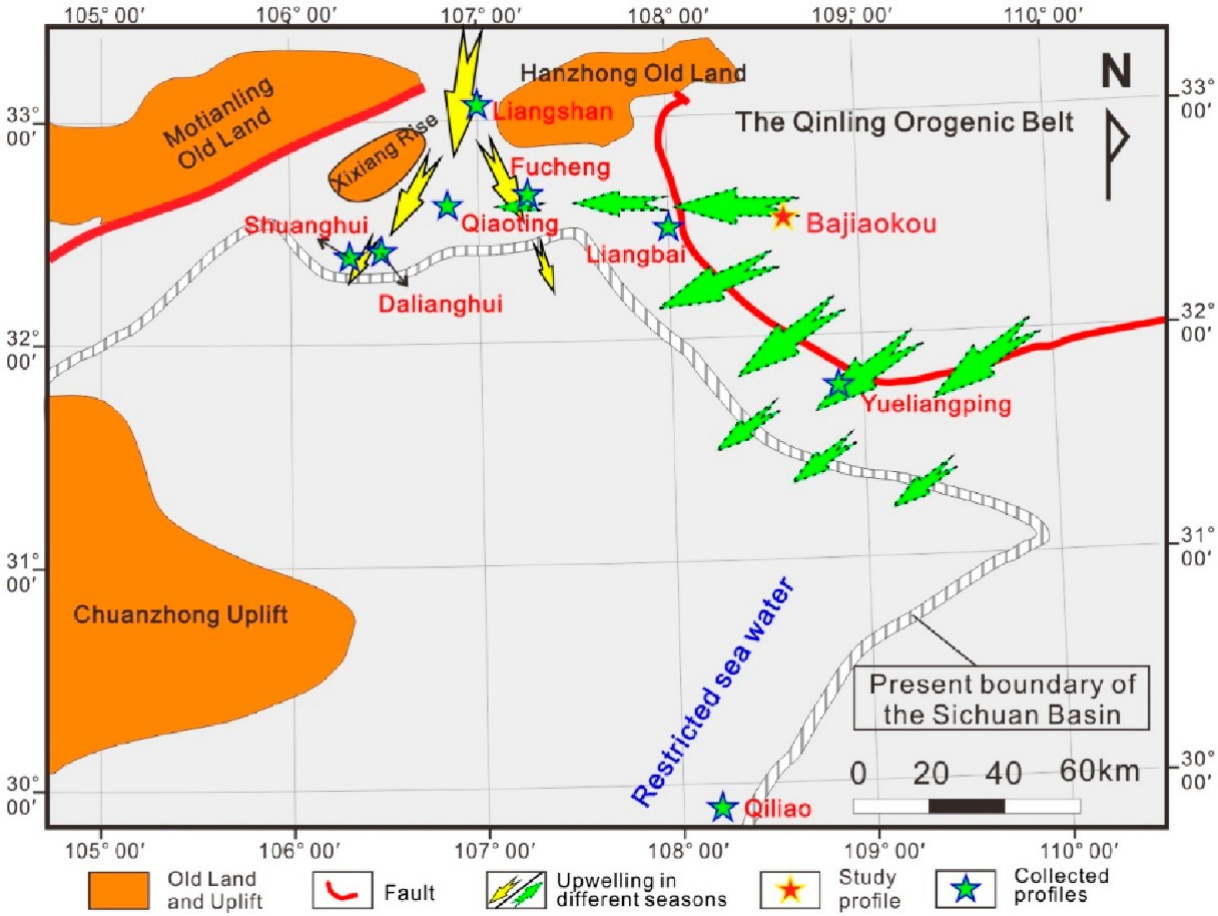
Impact range of upwelling currents during the sedimentary period of the Wufeng Formation.

Analysis of the water restricted degree of the Wufeng Formation was shown in Figure 9. The analysis of the Al–Co[EF] × Mn[EF] discriminant diagrams of the Wufeng Formation from the Dalianghui and Liangbai profiles in the Micangshan area suggested that samples were predominantly situated within the upwelling zone, indicating a primary influence of seasonal upwelling on Wufeng Formation sedimentation in this region. In cases where an offshore monsoon was sufficiently strong, it could induce nearshore upwelling. The presence of Wufeng Formation samples in both Qiaoting and Fucheng profiles within the upwelling zone implies the enduring effected of upwelling on these two sections. Despite the absence of elemental geochemical data for the Liangshan profile, Li^74^ (1997) determined through a study of the rock strata that the Wufeng Formation primarily consisted of black–gray calcareous mudstones and siltstone interbedded with radiolarian siliceous rocks. The hummocky cross-bedding observed in the siltstone was thought to be a result of distant storm deposition in a deep-water shelf area. It is hypothesized that the stratified radiolarian siliceous rocks developed in the profile were formed by seasonal continental margin upwelling.^74^ However, the samples from the Shuanghui profile’s Wufeng Formation were all distributed within a restricted marine environment, which may be related to the fact that the profile was located between the “Chuanzhong Uplift” and “Xixiang Rise” during the Wufeng Formation’s sedimentation period. Based on the above analysis, it is speculated that during the sedimentary period of the Wufeng Formation the Liangshan–Qiaoting–Dalianghui areas were located in the area affected by the upwelling currents generated by the monsoon from south to north (present direction). During the sedimentary period of the Wufeng Formation, the Liangbai–Fucheng–Qiaoting areas were located in the area affected by the upwelling currents generated by the monsoon from the west to the east (present direction). This leads to the formation of the areas in the Qiaoting and Fucheng regions where the two monsoons intersect, which are affected long-term by upwelling currents (Figure 10).

**Figure 11:**
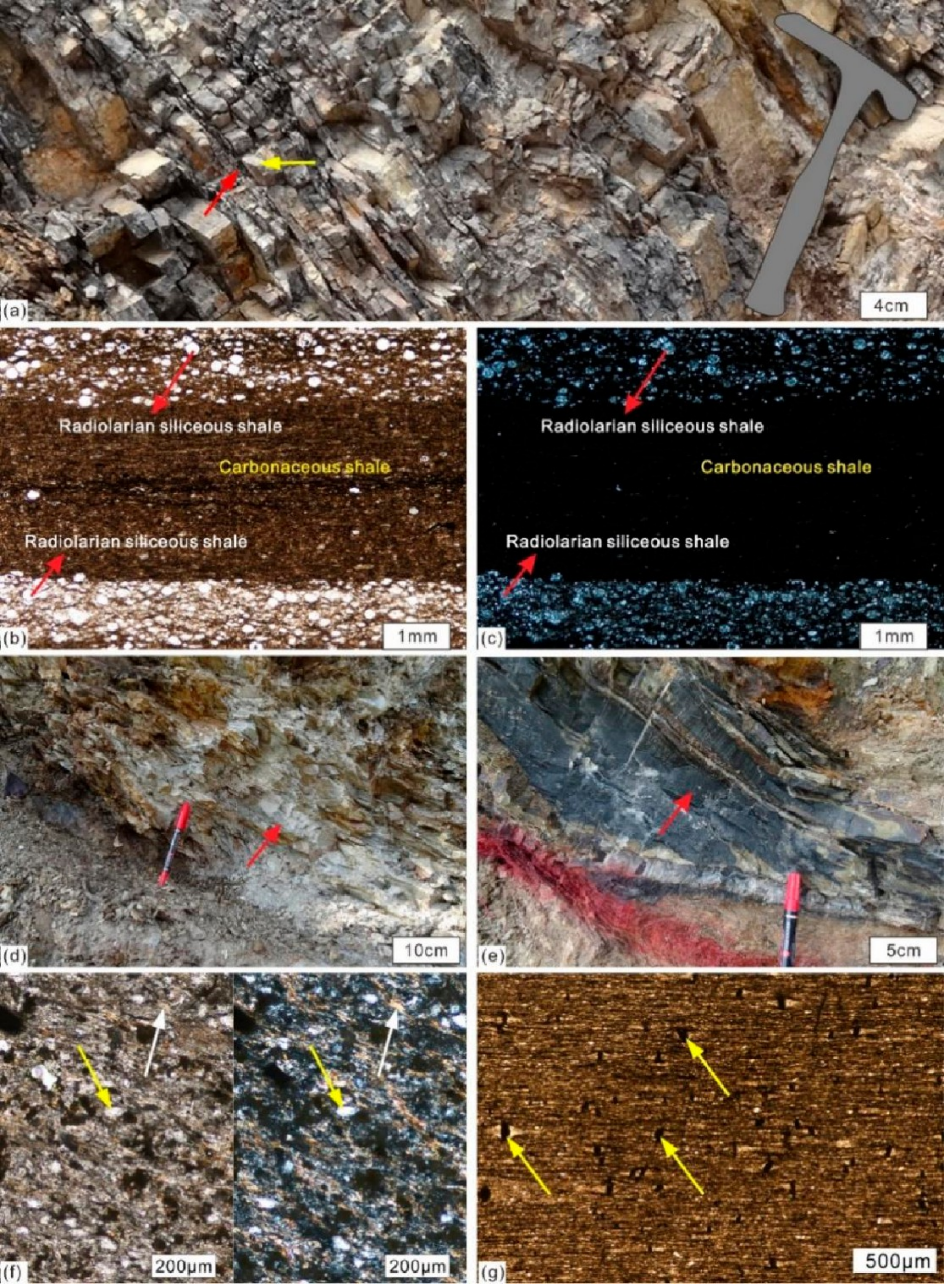
Typical outcrops and thin section photographs of the study area. Outcrop photographs: (a) Depicting a characteristic upwelling deposit rock assemblage of the Wufeng Formation in the Yueliangping profile, featuring carbonaceous shale (red arrow) interbedded with siliceous shale or siliceous rock (yellow arrow). Thin-section photographs: (b) Displaying a single polarized light microscopic photograph and (c) an orthogonal polarization microscopic photograph of thinly bedded radiolarian siliceous rock interlayered with carbonaceous shale from the Yueliangping profile. Outcrop photographs: (d) Gray white tuff (red arrow) of the Wufeng Formation in the Bajiaokou profile. (e) Silica shale (red arrow) of the lower part of the Longmaxi Formation in the Bajiaokou profile. Thin-section photographs: (f) sample BJK01 from the Bajiaokou profile, fine-grained glass debris sedimentary tuff; the left image shows single polarized light, and the right image shows orthogonal light, fine-grained debris (yellow arrow), and glass debris (white arrow). (g) Sample BJK14 from the Bajiaokou profile, siliceous shale, shows a large amount of pressure shadow structures with pyrite as the core (yellow arrow), and the shaded area is quartz.

These two different directions of monsoons also affect the entire Sichuan Basin and its surrounding areas. Located in the Dabashan region, the Yueliangping profile shows that part of the Wufeng Formation samples distributed within a restricted environment were identified by the Al–Co[EF] × Mn[EF] discriminant diagram, while a considerable number of samples fell within the upwelling area, indicating that the profile was influenced by seasonal upwelling currents during the deposition of the Wufeng Formation (Figure 9). Previous research on Palaeozoic and modern organic-rich rocks and sediments has demonstrated that well-developed sediments in upwelling zones exhibit unique compositions, including typical mineral–rock assemblages such as carbonaceous–siliceous mudstone assemblages and carbonaceous–siliceous–phosphorus mudstone assemblages.^75^ This study has identified similar rock assemblages in the thin sections of Wufeng Formation shale at the Yueliangping profile, including a carbonaceous–siliceous mudstone assemblage. Figure 11a illustrates that the carbonaceous shale of the Wufeng Formation at the Yueliangping profile is interbedded with siliceous shale or siliceous rock. The combination of thinly layered radiolarian siliceous shale and carbonaceous shale indicates that this area was influenced by upwelling currents during the sedimentation period of the Wufeng Formation (Figure 11b,c). The samples from the Wufeng Formation in the Bajiaokou profile on the southern margin of the Qinling Orogenic belt are all distributed in the upwelling current region (Figure 9), which is inferred to be affected by the upwelling current generated by the monsoon from southwest to northeast. What is different from the Yueliangping profile is that the Wufeng Formation in the Bajiaokou profile formed a combination of argillaceous shale, carbonaceous shale, and crystalline vitric tuff facies (Figure 11d,e,f), which is related to its acceptance of a large amount of volcanic tuff. The Qiliao profile, located in the eastern part of the Sichuan Basin, exhibits a clearly restricted water environment (Figure 9). It is speculated that this is because it is far away from the uplift in the basin interior and due to its location at the “Shizhu sedimentation center”,^37^ which forms a topography surrounded by other relatively high terrain.

#### 5.5.2 Water Restricted Degree of the Longmaxi Formation

**Figure 12:**
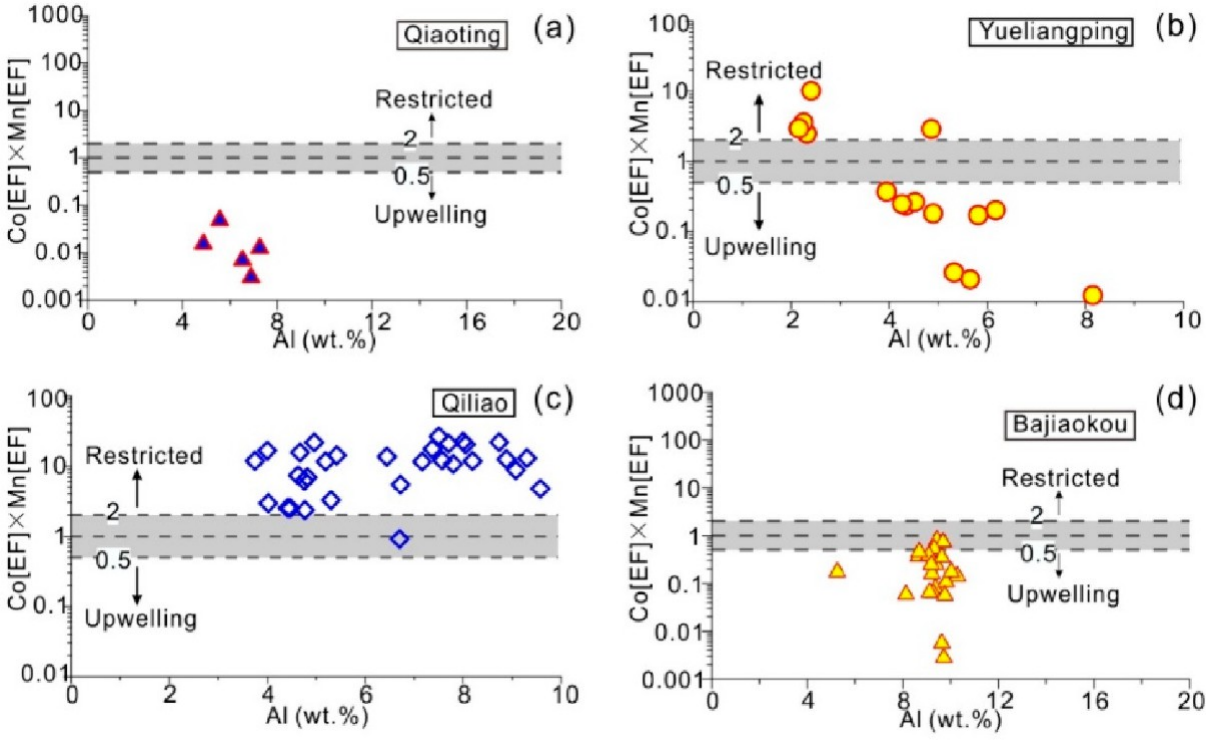
Co [EF] × Mn [EF] versus Al diagram of water mass restricted degree analysis in the Longmaxi Formation. (a) Qiaoting profile. (b) Yueliangping profile. (c) Qiliao profile. (d) Bajiaokou profile.

**Figure 13:**
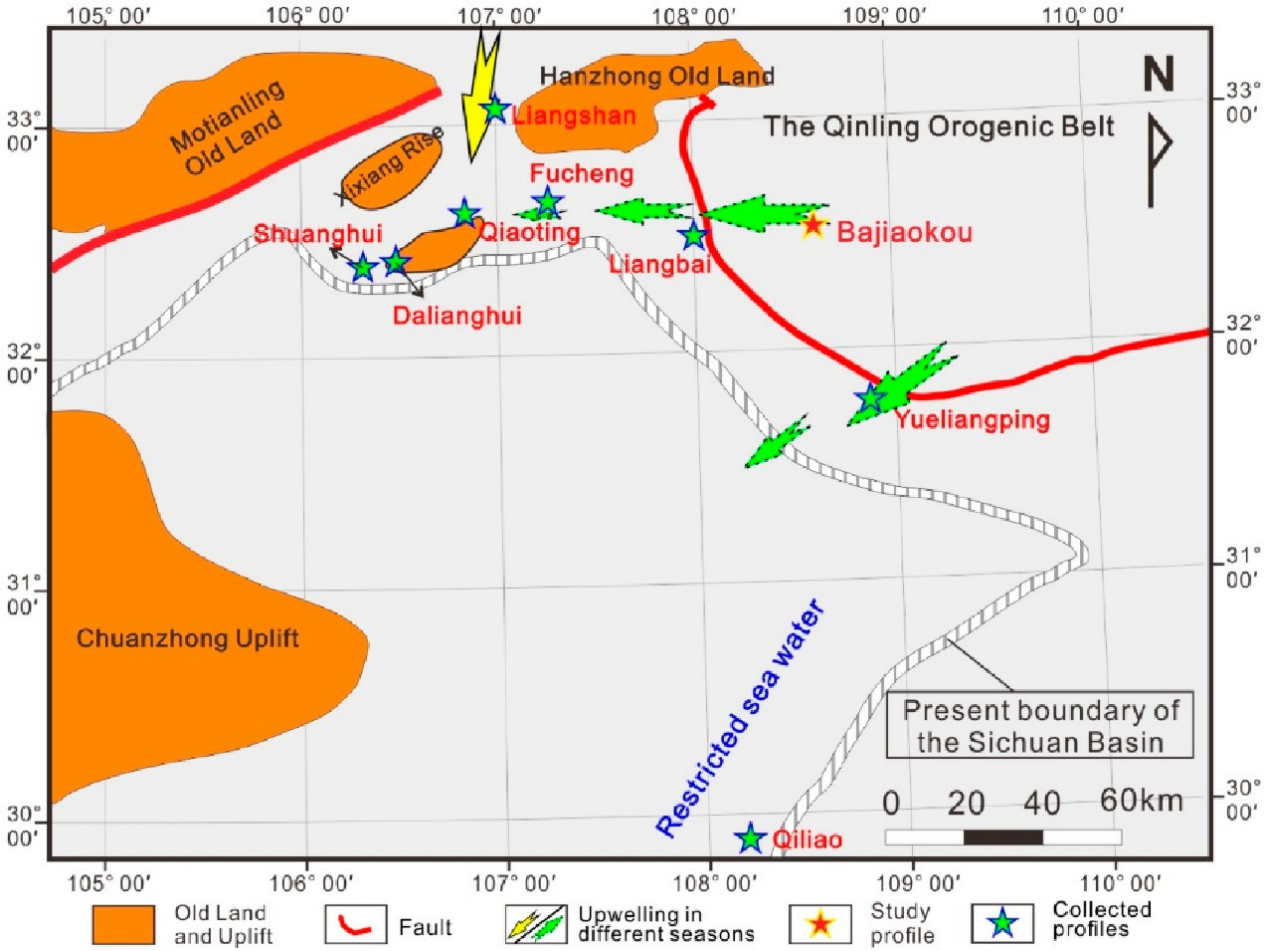
Impact range of upwelling currents during the sedimentary period of the Longmaxi Formation.

The analysis of the extent of ocean water restricted during the sedimentation period of the Longmaxi Formation was depicted in Figure 12. The geochemical data for the Longmaxi Formation at the front edge of the City of Micangshan were relatively scarce. The distribution range of upwelling currents during the sedimentary period of the “Black Shale Section” of the Longmaxi Formation in the Sichuan Basin and its surrounding areas is similar to that of the Wufeng Formation’s (Figure 13), but the intensity of upwelling currents is obviously lower than that of the Wufeng Formation based on the development of lithofacies combinations in various regions. This can also be proven by the fact that there is basically no siliceous lithofacies development in the Longmaxi Formation.^33^ The samples of Longmaxi Formation from the Qiaoting profile in the Micangshan area are all distributed in the upwelling current area of the Al–Co[EF] × Mn[EF] diagram (Figure 12). After experiencing the influence of the “Xixiang Raise”, the middle and upper parts of the “Black Shale Section” of the Longmaxi Formation were continuously affected by upwelling currents, forming a lithofacies assemblage of carbonaceous siliceous shale and argillaceous shale. The Longmaxi Formation at the Yueliangping and Bajiaokou profiles, as illustrated in Figure 12, exhibits similarities to the Wufeng Formation, with both being influenced by seasonal upwelling currents. Under the influence of upwelling currents, the Yueliangping profile in the northern slope area of the Sichuan Basin and the Bajiaokou profile in the southern margin of the Qinling have, respectively, formed siliceous shale, carbonaceous shale, and silty shale lithofacies assemblages, as well as carbonaceous siliceous shale and silty shale lithofacies assemblages. The samples of the Longmaxi Formation in the Qiliao profile in the eastern Sichuan Basin are all distributed in the restricted area of the Al–Co [EF] × Mn[EF] diagram (Figure 12). During the deposition period of the Longmaxi Formation, with the continuous subsidence of the “Shizhu sedimentation center”, the terrain decreased, and the degree of the restricted was enhanced compared with that of the Wufeng Formation, forming a lithofacies assemblage of carbonaceous shale and argillaceous shale.^37^

Based on the above analysis, it can be found that the upwelling currents almost affected the development of the Wufeng Formation and the “Black shale section” of the Longmaxi Formation in the northern margin of the Sichuan Basin and its surrounding areas. Only in some low-lying areas did it show a restricted water environment. The development of lithofacies can also correspond well to the intensity of upwelling currents. In the area with the strongest upwelling current in the northern Sichuan Basin, the siliceous shale lithofacies and even the siliceous lithofacies are widely developed, while in the area with relatively weak upwelling current in the eastern Sichuan Basin there are basically no siliceous lithofacies. In addition, the upwelling currents during the sedimentary period of the Wufeng Formation were significantly more active than those of the Longmaxi Formation, which may be related to the intensity of monsoons.

### 5.6 Organic Matter Accumulation Model of Shale and Shale in the Northern Sichuan and Southern Qinling Areas

The extent of oceanic water restriction is primarily influenced by sedimentary tectonic patterns, which subsequently impact the primary productivity of the ocean, redox conditions, and other factors, all of which play a pivotal role in regulating the accumulation of organic matter. Therefore, based on the analysis of the sedimentary environment of the Bajiaokou profile, combined with the previous research on the main controlling factors of organic matter enrichment in the Yueliangping profile in the northern margin of Sichuan Basin,^39^ the organic matter enrichment model of shale and shale in the study area is discussed.

Considering the significant disparities in detrital flux, redox conditions, and water body restrictions during the deposition periods of the Wufeng Formation and Longmaxi Formation, this study proposes two distinct mechanisms for the accumulation and preservation of organic matter in each formation.

#### 5.6.1 Accumulation Model of Organic Matter in the Wufeng Formation

**Figure 14:**
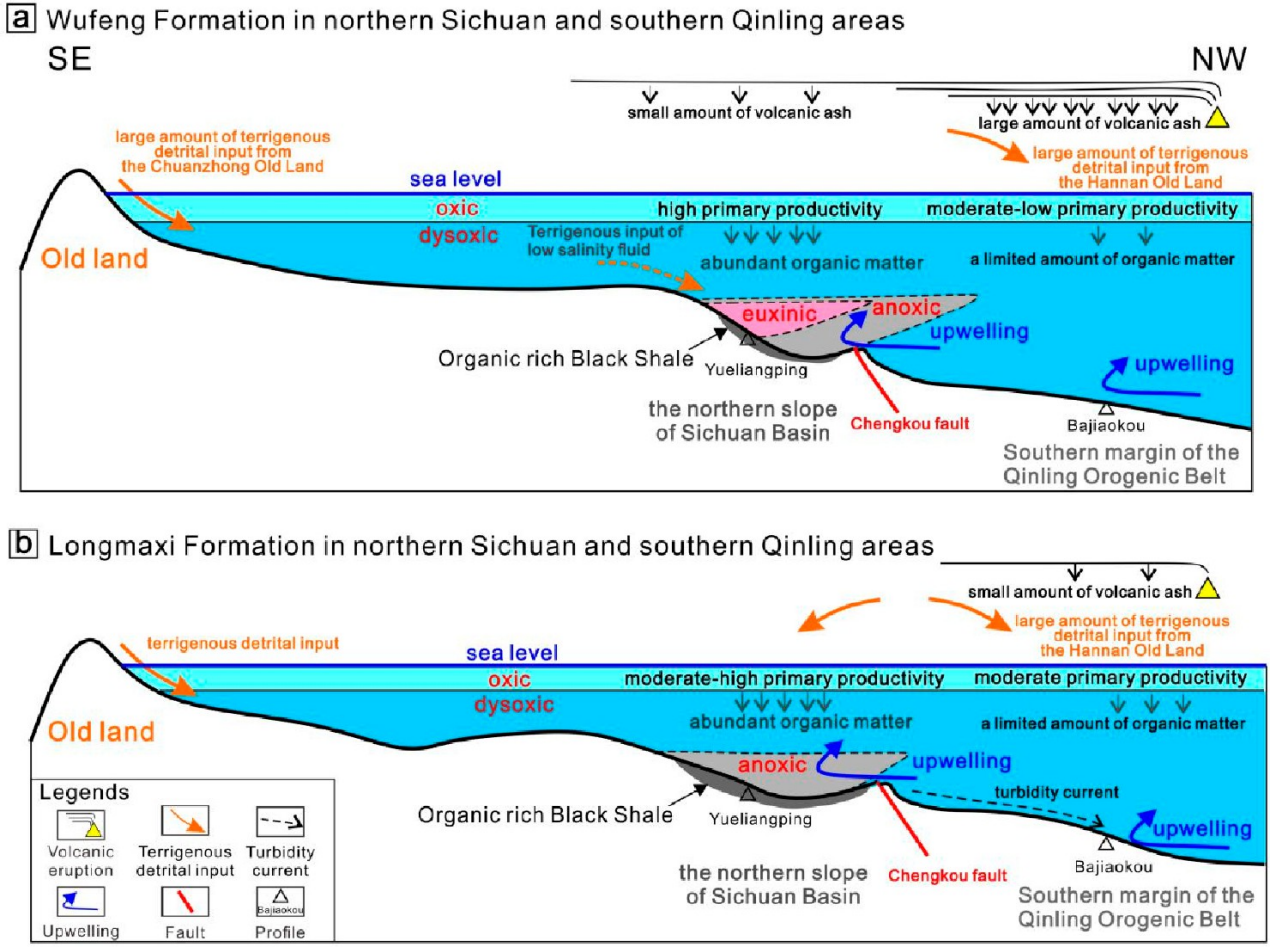
Schematic cross section illustrating the sedimentary tectonic pattern and organic matter accumulation model of the Wufeng–Longmaxi formations. (a) The Wufeng Formation in the northern Sichuan and southern Qinling areas. (b) The black shale member of the Longmaxi Formation in the northern Sichuan and southern Qinling areas. The locations of the profiles are indicated in Figure 1.

During the sedimentary period of the Wufeng Formation, the organic-rich black shale was most developed in the northern slope of the Sichuan Basin.^37^ Taking the Yueliangping profile in Chengkou as an example, during the deposition of the Wufeng Formation, the seasonal upwelling currents provided nutrients rich in Si, P, Ba, and other elements for the region. In addition, a large amount of volcanic ash was limited to the southern margin of the Qinling, only a small part of which was transported to the region (Figure 14). These small amounts of volcanic ash are altered by seawater during the deposition process, which could release some nutrients to promote the production of marine organisms, leading to a higher primary productivity in the region. The higher primary productivity not only provides a rich material basis for the enrichment of organic matter but also promotes the growth and reproduction of a large number of organisms, as well as the consumption of oxygen in the water body during the deposition of organic matter, resulting in anoxic in the lower part of the water body or even the euxinic marine conditions for vulcanization, which is very conducive to the preservation of organic matter.^76^ Li et al.^63^ (2015) proposed the “sulfide wedge” paleomarine chemical structure in the study of the Earth’s marine environment in the Early Cambrian and before. The formation of this structure is based on a sufficient supply of sulfate and organic matter. By analogy with modern oceans, Li et al.^63^ (2015) found that the sulfate content in modern seawater is high. Under sufficient sulfate supply, the supply of organic matter is the main factor restricting the development of marine sulfidation. In the modern open ocean, the sulfide water bodies are mainly distributed in the sea areas where the upwelling currents are developed because the nutrients carried by the upwelling current can promote the improvement of marine productivity. However, the high marine productivity can continue to reduce sulfate in seawater after consuming the dissolved oxygen and iron manganese oxides in seawater,^77^ thus forming sulfide water bodies. In the early ocean, the sulfate was rapidly consumed by the sulfate-reducing bacteria in a wide anoxic environment to form H_2_S, which was combined with Fe ions to form pyrite and was buried in large quantities, resulting in extremely low sulfate content in the early ocean.^78−81^ Therefore, the development of sulfur water bodies in the early ocean was controlled not only by the supply of marine organic matter but also by the availability of marine sulfate.^63^ In the areas of the northern Sichuan Basin and the southern Qinling in this study, a large amount of terrigenous debris and oxygen-rich water from the Chuanzhong Uplift and Hanzhong Oldland led to the overall dysoxic condition of the water body in this area. The sulfate in the ocean may not be rapidly consumed by sulfate-reducing bacteria; therefore, the most likely factor restricting the formation of sulfide water is the supply of organic matter. In the Yueliangping profile situated on the northern slope of the Sichuan Basin, the high primary productivity during the Wufeng Formation sedimentation period resulted in a substantial accumulation of organic matter, providing a robust material foundation for organic matter enrichment. The profile is positioned at the confluence of terrestrial input and coastal upwelling. The mixing of fluids with varying salinities from terrestrial and marine sources in seawater results in the formation of a wedge-shaped pattern of sulfide-rich water extending from the coast to the ocean. This structure is more developed in ancient oceanic settings and is known as the “sulfide wedge” paleomarine chemical structure,^78,82^ as illustrated in Figure 14a. Although some organic matter is consumed during the formation of the “sulfide wedge”, the development of this structure greatly contributes to the preservation of organic matter. It is due to the presence of this structure that the Yueliangping profile has developed the carbonaceous siliceous shale facies with a significant accumulation of organic matter, establishing “a model of organic matter enrichment cooperatively maintained by the high primary productivity and the sulfide wedge under the action of upwelling currents”.

The Bajiaokou profile, located in the southern Qinling Orogenic Belt, had a warm and humid climate during the deposition period of the Wufeng Formation. In the lower part of the Wufeng Formation, it was mainly deposited with gray argillaceous shale, tuffaceous shale lithofacies, and crystalline vitric tuff lithofacies. Chen et al.^83^ (2018) studied the volcanic activity of the Wufeng–Longmaxi formations in the Upper Yangtze area, and proposed that the area of volcanic ash scattering after the volcanic activity was large enough, and the dissolution of volcanic ash in the ocean could provide rich nutrients for organisms in the water body. However, compared with the thin tuff layers developed in the Yueliangping profile in the northern part of the Sichuan Basin, the tuff layers in the Bajiaokou profile are significantly more and thicker. Although the upwelling currents were well developed during the deposition of the Wufeng Formation, this concentrated accumulation of volcanic ash was not conducive to the growth and reproduction of marine organisms,^84^ resulting in low primary productivity in the ocean. During this period, the clastic flux, which was dominated by clay minerals, was high. The water body was in the condition of the oxic-dysoxic. The organic matter in the sediment was not accumulated with a low content of the TOC. During this period, the low primary productivity of seawater and the condition of the oxic-dysoxic were the main factors leading to the nonenrichment of organic matter (Figure 14a).

#### 5.6.2 Accumulation Model of Organic Matter in the Longmaxi Formation

During the sedimentary period of the Longmaxi Formation, the organic-rich black shale was still the most developed in the northern slope of the Sichuan Basin. The Yueliangping profile continued the organic matter enrichment pattern in the sedimentary period of the Wufeng Formation, but the structure of the “sulfide wedge” did not develop in the sedimentary period of the Longmaxi Formation, which may be caused by the limitation of the sulfate supply in the terrigenous input. However, seasonal upwelling brings rich nutrients to the surface waters, increasing ocean primary productivity and forming moderate to high primary productivity. Meanwhile, a large amount of organic matter in the settling process consumes oxygen in the water, leading to the formation of anoxic conditions in the lower part of the water body, which is conducive to the preservation of organic matter. Finally, in this area, organic-rich carbonaceous argillaceous shale and the carbonaceous silty shale lithofacies were formed, which is “a model of organic matter enrichment synergistically promoted by the high primary productivity and the anoxic bottom water under the action of upwelling currents” (Figure 14b).

The Bajiaokou profile, located in the southern part of the Qinling Orogenic Belt, showed a decrease in the level of volcanic ash during the sedimentation period of the Longmaxi Formation. The large amount of terrestrial debris and oxygen-rich water brought by the Hanzhong Old Land and deep-water turbidity currents led to an overall dysoxic water body condition in this area. Compared with the Wufeng Formation, the development of seasonal upwelling currents and a small amount of volcanic ash supply have increased the primary productivity in the Longmaxi Formation. However, the formed organic matter was destroyed by oxidation in the slow sedimentation process and finally formed the gray–black siliceous shale and argillaceous shale lithofacies with slight enrichment of organic matter. During this period, the moderate primary productivity provided a good material basis for the enrichment of organic matter, but the high terrigenous detrital flux and oxic–dysoxic conditions were the main factors leading to the slight enrichment of organic matter (Figure 14b).

## 6 Conclusions

This research elucidates the environmental conditions during the sedimentary period of the Wufeng–Longmaxi formations in the southern margin of the Qinling Orogenic Belt and establishes the shale sedimentary model. The following conclusions can be drawn from this work:

(1) From the Late Ordovician to the Early Silurian, the southern Qinling area generally had a warm and humid climate. During the shale deposition period of Wufeng–Longmaxi formations, the marine primary productivity in the southern Qinling area is generally low to moderate. The upwelling current is widely developed in the northern margin of the Sichuan Basin and the southern margin of the Qinling area, and the water environment is restricted only in some low-lying areas.
(2) During the sedimentary period of the Wufeng Formation in the study area, the concentrated accumulation of a large amount of volcanic ash resulted in low marine primary productivity. During this period, the terrigenous detrital flux was high and dominated by the clay minerals, the water body presenting the condition of oxic–dysoxic, and the organic matter was not enriched in the sediment.
(3) During the sedimentary period of the Longmaxi Formation in the study area, the development of seasonal upwelling currents and a small amount of volcanic ash supply increased the primary productivity to moderate, which provided a good material basis for the enrichment of organic matter, but the high detritus flux and the water body condition of oxic–dysoxic resulted in the slight enrichment of organic matter.

## Supporting Information Available

Supp. Table 1. Contents of TOC and major-element oxides of samples in the Bajiaokou profile (in wt %). Supp. Table 2. The trace elements data and the chemical index of alteration (CIA) for the samples in the Bajiaokou profile. Supp. Table 3. Number of geochemical test samples of major and trace elements and sources of cited data. Supp. Table 4. Content of Al, Mn, and Co elements in samples from the Wufeng-Longmaxi formations in the Bajiaokou profile. Supp. Table 5. Content of Al, Mn, and Co elements in samples from the Wufeng-Longmaxi formations in the Yueliangping and Qiaoting profiles. Supp. Table 6. Content of Al, Mn, and Co elements in samples from the Wufeng Formation in the Liangbai, Fucheng, Dalianghui, and Shuanghui profiles. Supp. Table 7. Content of Al, Mn, and Co elements in samples from the Wufeng-Longmaxi formations in the Qiliao profile (PDF)

## Abbreviations

TOC: total organic carbon
PAAS: post-Archean Australian shale
CIA: chemical index of alteration
