# Supplementary material for: Elemental Geochemical Characteristics of Shales from Wufeng–Longmaxi Formations in the Southern Margin of the Qinling Orogenic Belt, China: Implications for Depositional Controls on Organic Matter
Source: ACS Omega. 2024 Jul 15;9(29):31488–507. doi: 10.1021/acsomega.4c01129 (PMC11270573; doi:10.1021/acsomega.4c01129)
Supplement: Supplementary file 1 — ao4c01129_si_001.pdf [file ao4c01129_si_001.pdf]

Supporting Information

Elemental geochemical characteristics of Shales from Wufeng−Longmaxi Formations in the southern margin of the Qinling Orogenic Belt, China: Implications for depositional controls on organic matter

Bin Xiao[a,*], Dongxu Guo[a], Zhongying Zhao[b], Shuzhen Xiong[c], Mingfei Feng[a], Zhonghai Zhao[a], Sheng Li[d]

a. College of Mining, Liaoning Technical University, Fuxin 123000, China

b. PetroChina Research Institute of Petroleum Exploration & Development, Beijing 100083, China

c. College of Environmental Science and Engineering, Liaoning Technical University, Fuxin 123000, China

d. Liaoning Geology Engineering Vocational College, Dandong 118000, China

Supp. Table 1. Contents of TOC and major-element oxides of samples in Bajiaokou profile (in wt.%)

Sample	Lithology	TOC	SiO_2	TiO_2	Al_2O_3	TFe_2O_3	MnO	MgO	CaO	Na_2O	K_2O	P_2O_5
BJK01	Tuff	0.11	73.27	2.59	14.49	1.62	0.01	1.01	0.05	0.32	4.26	0.26
BJK02	Argillaceous shale	0.26	72.23	0.95	13.88	4.20	0.03	1.58	0.19	0.77	3.10	0.12
BJK03	Siliceous shale	0.17	73.72	1.61	13.06	2.61	0.02	1.31	0.86	0.34	3.30	0.19
BJK04	Siliceous shale	0.15	61.75	0.84	19.52	6.10	0.04	2.18	0.12	0.61	4.17	0.10
BJK05	Argillaceous shale	0.28	64.31	0.82	18.08	5.62	0.03	1.77	0.11	0.17	4.15	0.10
BJK06	Tuff	0.12	68.62	2.73	17.27	1.70	0.01	1.17	0.04	0.20	5.20	0.17
BJK07	Tuff	0.11	70.48	2.47	15.89	1.83	0.01	1.15	0.24	0.54	4.43	0.19
BJK08	Carbonaceous shale	1.02	63.16	0.82	17.41	6.00	0.05	2.59	0.25	0.23	4.00	0.14
BJK09	Carbonaceous siliceous shale	0.96	55.73	1.43	17.82	8.13	0.05	2.46	0.94	0.17	4.33	0.23
BJK10	Siliceous shale	0.30	63.61	0.80	17.67	6.65	0.05	2.73	0.02	0.22	3.96	0.13
BJK11	Carbonaceous siliceous shale	0.55	65.44	0.83	16.35	5.68	0.05	2.47	0.20	0.48	3.76	0.10
BJK12	Carbonaceous siliceous shale	0.55	59.15	1.35	18.31	6.82	0.04	2.21	0.61	0.09	4.33	0.21
BJK13	Argillaceous shale	0.43	63.25	0.80	17.76	6.14	0.05	2.73	0.12	0.40	4.01	0.14
BJK14	Carbonaceous siliceous shale	0.62	62.67	0.95	17.48	7.01	0.04	2.53	0.28	0.21	3.96	0.13
BJK15	Carbonaceous siliceous shale	0.76	62.34	1.33	19.43	4.04	0.02	1.22	0.02	0.04	4.49	0.16
BJK16	Carbonaceous siliceous shale	0.68	60.64	1.20	17.58	6.90	0.05	2.57	0.63	0.38	4.18	0.26
BJK17	Silty shale	0.40	64.17	0.83	17.22	6.71	0.04	2.51	0.10	0.05	3.91	0.14
BJK18	Carbonaceous siliceous shale	0.51	63.69	0.93	17.15	6.28	0.05	2.50	0.24	0.40	3.94	0.15
BJK19	Argillaceous shale	0.19	69.11	0.77	15.36	4.93	0.03	1.83	0.17	0.17	3.66	0.10
BJK20	Argillaceous shale	0.24	61.72	0.86	18.54	7.02	0.04	2.47	0.18	0.34	4.24	0.17
BJK21	Argillaceous shale	0.23	60.78	0.89	18.95	6.43	0.05	2.78	0.16	0.43	4.43	0.15
BJK22	Silty carbonaceous shale	0.68	60.67	1.06	18.21	7.02	0.04	2.35	0.28	0.23	4.31	0.12
BJK23	Silty shale	0.06	78.59	0.94	9.92	3.93	0.03	1.46	0.43	0.45	1.89	0.18
BJK24	Silty carbonaceous shale	0.94	62.62	1.07	18.20	5.92	0.02	1.50	0.14	0.26	4.07	0.09
BJK25	Silty carbonaceous shale	0.76	63.29	1.05	16.41	5.90	0.04	2.23	0.48	0.41	3.75	0.16
BJK26	Tuff	0.10	64.20	2.96	19.73	1.90	0.01	1.30	0.03	0.00	5.98	0.25
BJK27	Carbonaceous shale	2.08	63.74	2.78	18.36	1.74	0.01	1.24	0.06	0.00	5.58	0.49
BJK28	Silty carbonaceous shale	0.94	63.81	0.83	17.35	5.89	0.05	2.64	0.30	0.25	3.96	0.12
BJK29	Silty carbonaceous shale	0.46	62.48	1.00	18.44	5.98	0.04	2.49	0.18	0.34	4.32	0.16

Supp. Table 2. The trace elements data and the chemical index of alteration (CIA) for the samples in Bajiaokou profile

Sample	Zr (μg/g)	V (μg/g)	U (μg/g)	Th (μg/g)	Cr (μg/g)	Sc (μg/g)	Mo (μg/g)	P/Ti	Ba/Al (10^{-4})	Si_{XS} (wt.%)	CIA	CIA_{corr}
BJK01	184.8	194.5	3.34	13.55	157.2	15.57	11.89	0.07	450.82	10.35	73.44	83.64
BJK02	181.3	130.5	3.14	12.76	84.1	13.94	2.85	0.09	332.74	10.87	73.56	78.07
BJK03	226.7	130	2.75	12.76	91.99	13.76	2.97	0.08	372.75	12.91	73.52	80.29
BJK04	231.6	156.2	3.37	16.56	109.1	20.27	0.74	0.09	317.15	-3.32	77.24	82.05
BJK05	194.2	156.1	3.36	15.46	102.9	19.29	2.75	0.08	337.81	0.25	78.40	84.94
BJK06	369.7	236.6	4.80	20.98	177.4	21.01	5.13	0.05	469.60	3.60	74.07	85.19
BJK07	554.9	208.4	4.19	17.05	179	21.19	4.90	0.05	439.44	6.73	72.20	80.54
BJK08	199.3	175.3	4.21	16.09	102	17.92	4.08	0.12	349.21	0.82	77.14	83.26
BJK09	283.1	212.3	4.42	15	98.43	20.45	7.98	0.11	407.69	-3.33	77.28	84.60
BJK10	198.4	143.3	3.01	14.78	107.5	19.32	0.83	0.12	355.78	0.60	78.97	85.21
BJK11	194.5	151.1	3.47	15.22	102.8	18.49	2.12	0.09	375.27	3.63	75.79	81.50
BJK12	214.7	206.2	3.65	13.79	98.83	20.88	5.54	0.11	405.18	-2.54	78.53	85.76
BJK13	197.2	143.8	3.47	15.48	107.7	18.78	1.55	0.13	357.83	0.29	77.28	83.12
BJK14	225.3	166.8	3.98	14.98	119.1	19.13	5.80	0.10	357.49	0.47	77.27	83.18
BJK15	292.8	216.5	4.72	15.28	105.2	21.32	6.06	0.09	385.65	-2.88	79.59	86.72
BJK16	218.7	189.9	3.74	14.57	102.3	19.79	6.39	0.16	383.91	-0.64	73.58	79.21
BJK17	216.5	158.6	3.64	15.15	105.9	18.66	2.12	0.12	354.65	1.60	79.22	85.83
BJK18	239.7	173.6	3.91	15.97	99.67	19.26	2.28	0.12	351.27	1.49	76.15	81.96
BJK19	174.6	149.4	2.86	13.63	92.26	16.69	2.17	0.10	351.69	6.97	77.07	83.90
BJK20	210	152	3.31	16.05	106.2	19.39	0.66	0.14	338.56	-1.71	77.15	83.22
BJK21	209.9	163.7	3.17	15.97	109.9	21.12	0.59	0.13	345.70	-2.83	76.57	82.87
BJK22	250.8	184.6	4.79	15.84	108.5	20.75	6.69	0.08	371.50	-1.66	76.64	83.20
BJK23	201.3	79.53	2.82	13.55	76.28	9.757	4.94	0.14	293.77	20.34	73.56	75.66
BJK24	274.6	178.8	4.23	16.1	108.1	21.03	7.22	0.06	363.22	-0.74	78.14	84.06
BJK25	249.8	182.7	3.40	13.97	95.61	19.49	3.10	0.11	366.70	2.53	74.47	79.66
BJK26	366.2	332.9	9.78	26.74	83.25	22.34	9.30	0.06	531.43	-2.52	75.13	86.98
BJK27	339.3	305.5	10.11	24.85	84.46	20.26	14.39	0.13	525.37	-0.47	74.88	86.70
BJK28	209.1	169.7	4.17	16.02	101.1	17.18	3.85	0.11	357.50	1.22	76.75	82.65
BJK29	225.6	172.2	3.70	15.8	109.6	21.11	2.83	0.11	392.31	-1.20	76.78	83.20

Supp. Table 3. Number of geochemical test samples of major and trace elements and sources of cited data.

Profile	Stratigraphic position	Al(wt.%)	Mn(wt.%)	Co(ppm)	Mo(ppm)	TOC(wt.%)
Bajiaokou profile	Wufeng Formation + Longmaxi Formation	4+21a	4+21a	4+21a	---	4+21a
Shuanghui profile	Wufeng Formation	6b	6b	6b	---	---
Dalianghui profile	Wufeng Formation	4b	4b	4b	---	---
Fucheng profile	Wufeng Formation	9b	9b	9b	---	---
Liangbai profile	Wufeng Formation	4b	4b	4b	---	---
Qiliao profile	Wufeng Formation + Longmaxi Formation	7+31c	7+31c	7+31c	---	7+31c
Yueliangping profile	Wufeng Formation + Longmaxi Formation	10+15d	10+15d	10+15d	---	12+15d
Qiaoting profile	Wufeng Formation + Longmaxi Formation	3+5d	3+5d	3+5d	---	3+5d

a: The measured data in this paper; b: Sun et al., (2016); c: Li et al., (2017); d: Xiao et al., (2022);.

Supp. Table 4. Content of Al, Mn, and Co elements in samples from the Wufeng-Longmaxi formations in the Bajiaokou profile

Profile	Sample No.	Formation	Al(wt.%)	Mn(wt.%)	Co(ppm)
Bajiaokou profile	BJK02	O_3w	7.35	0.023	5.09
	BJK03	O_3w	6.91	0.012	1.95
	BJK04	O_3w	10.33	0.032	5.72
	BJK05	O_3w	9.57	0.025	9.33
	BJK08	S_1l	9.22	0.038	6.46
	BJK09	S_1l	9.43	0.037	36.03
	BJK10	S_1l	9.35	0.039	3.28
	BJK11	S_1l	8.65	0.036	14.11
	BJK12	S_1l	9.69	0.032	39.12
	BJK13	S_1l	9.40	0.039	10.19
	BJK14	S_{1l}	9.25	0.033	3.02
	BJK15	S_{1l}	10.28	0.012	21.96
	BJK16	S_{1l}	9.30	0.039	21.62
	BJK17	S_1l	9.11	0.032	3.05
	BJK18	S_1l	9.08	0.039	14.79
	BJK19	S_1l	8.13	0.025	2.82
	BJK20	S_1l	9.81	0.033	5.68
	BJK21	S_1l	10.03	0.042	7.69
	BJK22	S_1l	9.64	0.035	17.14
	BJK23	S_1l	5.25	0.020	4.44
	BJK24	S_1l	9.63	0.015	0.62
	BJK25	S_1l	8.68	0.033	18.70
	BJK27	S_1l	9.72	0.006	0.83
	BJK28	S_1l	9.18	0.037	10.34
	BJK29	S_1l	9.76	0.031	3.13

Supp. Table 5. Content of Al, Mn, and Co elements in samples from the Wufeng-Longmaxi formations in the Yueliangping and Qiaoting profiles

Profile	Sample No.	Formation	Al(wt.%)	Mn(wt.%)	Co(ppm)
Yueliangping profile	YLP01	O_3w	0.89	0.031	30.35
	YLP02	O_3w	1.97	0.008	7.92
	YLP03	O_3w	1.54	0.023	3.13
	YLP04	O_3w	2.12	0.008	1.66
	YLP05	O_3w	2.45	0.023	10.11
	YLP06	O_3w	2.18	0.023	14.03
	YLP07	O_3w	2.36	0.008	16.22
	YLP08	O_3w	1.85	0.023	11.10
	YLP10	O_3w	5.57	0.008	12.31
	YLP11	O_3w	3.32	0.008	11.30
	YLP12	O_3w	1.48	0.248	5.11
	YLP13	S_1l	4.34	0.008	10.46
	YLP14	S_1l	2.32	0.015	16.05
	YLP15	S_1l	2.25	0.015	21.59
	YLP16	S_1l	4.89	0.008	10.33
	YLP17	S_1l	2.40	0.015	70.21
	YLP18	S_1l	2.15	0.015	16.41
	YLP19	S_1l	3.94	0.008	13.72
	YLP20	S_1l	4.52	0.008	12.82
	YLP21	S_1l	5.81	0.008	13.79
	YLP22	S_1l	4.26	0.008	10.66
	YLP23	S_1l	5.32	0.008	1.76
	YLP24	S_1l	4.85	0.07	18.12
	YLP25	S_1l	8.15	0.008	1.96
	YLP26	S_1l	5.65	0.008	1.58
	YLP27	S_1l	6.17	0.008	18.29
Qiaoting profile	QT01	O_3w	8.78	0.057	28.53
	QT02	O_3w	7.14	0.003	4.06
	QT03	O_3w	4.88	0.003	1.10
	QT04	S_1l	6.53	0.005	1.00
	QT05	S_1l	6.89	0.003	0.85
	QT06	S_1l	4.87	0.005	1.45
	QT07	S_1l	5.56	0.006	4.44
	QT08	S_1l	7.25	0.006	1.96

Supp. Table 6. Content of Al, Mn, and Co elements in samples from the Wufeng Formation in the Liangbai, Fucheng, Dalianghui, and Shuanghui profiles

Profile	Sample No.	Formation	Al(wt.%)	Mn(wt.%)	Co(ppm)
Liangbai profile	WXP-B7	O_3w	7.51	0.008	11.50
	WXP-B8	O_3w	5.03	0.062	52.40
	XLP-B3	O_3w	9.40	0.015	9.23
	XLP-B4-1	O_3w	1.25	0.031	20.70
	XLP-B5	O_3w	11.41	0.070	24.20
	XLP-B6	O_3w	8.68	0.015	7.96
Fucheng profile	NFP-B3	O_3w	0.95	0.008	0.37
	NFP-B4	O_3w	4.67	0.008	0.75
	NFP-B6	O_3w	2.78	0.008	0.32
	NFP-B7	O_3w	1.07	0.008	0.46
	NFP-B9	O_3w	2.72	0.008	0.82
	NFP-B11	O_3w	2.86	0.008	1.60
	NFP-B13	O_3w	3.12	0.008	2.14
	NFP-B16	O_3w	1.57	0.008	3.81
	NFP-B17	O_3w	6.60	0.031	41.90
Dalianghui profile	DLHP-B2	O_3w	9.60	0.023	20.20
	DLHP-B4	O_3w	5.34	0.705	57.70
	DLHP-B6	O_3w	8.57	0.046	33.20
	DLHP-B9	O_3w	5.68	0.015	6.53
Shuanghui profile	SHP-B2	O_3w	8.65	0.070	45.30
	SHP-B5	O_3w	1.06	0.008	2.69
	SHP-B8	O_3w	0.79	0.008	0.92
	SHP-B11	O_3w	1.71	0.031	7.38
	SHP-B15	O_3w	0.51	0.015	1.38
	SHP-B16	O_3w	5.27	0.310	12.70

Supp. Table 7. Content of Al, Mn, and Co elements in samples from the Wufeng-Longmaxi formations in the Qiliao profile

Profile	Sample No.	Formation	Al(wt.%)	Mn(wt.%)	Co(ppm)
Qiliao profile	SZ-05	O_3W1	4.92	1.294	2.00
	SZ-06	O_3W1	3.62	0.434	1.00
	SZ-08	O_3W1	4.21	0.565	8.00
	WL-01	O_3W1	9.43	1.596	4.00
	WL-02	O_3W1	8.19	1.023	7.00
	WL-03	O_3W1	6.79	0.821	7.00
	WL-04	O_3W1	6.94	0.844	5.00
	WL-05	S_{1l}	4.02	0.442	2.00
	WL-06	S_{1l}	5.19	0.581	10.00
	WL-07	S_{1l}	3.75	0.387	8.00
	WL-08	S_{1l}	4.76	0.519	5.00
	WL-09	S_{1l}	4.67	0.527	12.00
	WL-10	S_{1l}	4.00	0.411	12.00
	WL-12	S_1l	4.42	0.457	2.00
	WL-13	S_1l	4.77	0.496	2.00
	WL-14	S_1l	4.47	0.465	2.00
	WL-15	S_1l	5.42	0.596	13.00
	WL-16	S_1l	4.82	0.496	6.00
	WL-17	S_1l	4.96	0.550	18.00
	WL-19	S_1l	4.63	0.488	6.00
	WL-20	S_1l	5.30	0.565	3.00
	SZ-17	S_1l	6.44	0.697	15.00
	SZ-18	S_1l	6.72	0.759	6.00
	SZ-19	S_1l	6.70	0.759	1.00
	SZ-21	S_1l	7.16	0.689	16.00
	SZ-26	S_1l	7.37	1.038	17.00
	SZ-32	S_1l	7.57	0.914	14.00
	SZ-37	S_1l	7.70	1.534	15.00
	SZ-46	S_1l	8.19	1.224	12.00
	SZ-53	S_1l	7.79	0.860	14.00
	SZ-54	S_1l	9.07	1.216	11.00
	SZ-56	S_1l	8.88	1.526	12.00
	SZ-62	S_1l	7.50	1.735	16.00
	SZ-69	S_1l	8.00	1.898	14.00
	SZ-79	S_1l	9.30	1.387	15.00
	SZ-80	S_1l	8.73	1.627	19.00
	SZ-85	S_1l	8.05	1.286	19.00
	SZ-88	S_1l	9.57	1.154	7.00
